# Dynamic sumoylation of promoter-bound general transcription factors facilitates transcription by RNA polymerase II

**DOI:** 10.1371/journal.pgen.1009828

**Published:** 2021-09-29

**Authors:** Mohammad S. Baig, Yimo Dou, Benjamin G. Bergey, Russell Bahar, Justin M. Burgener, Marjan Moallem, James B. McNeil, Akhi Akhter, Giovanni L. Burke, Veroni S. Sri Theivakadadcham, Patricia Richard, Damien D’Amours, Emanuel Rosonina

**Affiliations:** 1 Department of Biology, York University, Toronto, Ontario, Canada; 2 Stellate Therapeutics, New York, New York, United States of America; 3 Ottawa Institute of Systems Biology, Department of Cellular and Molecular Medicine, University of Ottawa, Ottawa, Ontario, Canada; Walter and Eliza Hall Institute of Medical Research, AUSTRALIA

## Abstract

Transcription-related proteins are frequently identified as targets of sumoylation, including multiple subunits of the RNA polymerase II (RNAPII) general transcription factors (GTFs). However, it is not known how sumoylation affects GTFs or whether they are sumoylated when they assemble at promoters to facilitate RNAPII recruitment and transcription initiation. To explore how sumoylation can regulate transcription genome-wide, we performed SUMO ChIP-seq in yeast and found, in agreement with others, that most chromatin-associated sumoylated proteins are detected at genes encoding tRNAs and ribosomal proteins (RPGs). However, we also detected 147 robust SUMO peaks at promoters of non-ribosomal protein-coding genes (non-RPGs), indicating that sumoylation also regulates this gene class. Importantly, SUMO peaks at non-RPGs align specifically with binding sites of GTFs, but not other promoter-associated proteins, indicating that it is GTFs specifically that are sumoylated there. Predominantly, non-RPGs with SUMO peaks are among the most highly transcribed, have high levels of TFIIF, and show reduced RNAPII levels when cellular sumoylation is impaired, linking sumoylation with elevated transcription. However, detection of promoter-associated SUMO by ChIP might be limited to sites with high levels of substrate GTFs, and promoter-associated sumoylation at non-RPGs may actually be far more widespread than we detected. Among GTFs, we found that TFIIF is a major target of sumoylation, specifically at lysines 60/61 of its Tfg1 subunit, and elevating Tfg1 sumoylation resulted in decreased interaction of TFIIF with RNAPII. Interestingly, both reducing promoter-associated sumoylation, in a sumoylation-deficient Tfg1-K60/61R mutant strain, and elevating promoter-associated SUMO levels, by constitutively tethering SUMO to Tfg1, resulted in reduced RNAPII occupancy at non-RPGs. This implies that dynamic GTF sumoylation at non-RPG promoters, not simply the presence or absence of SUMO, is important for maintaining elevated transcription. Together, our findings reveal a novel mechanism of regulating the basal transcription machinery through sumoylation of promoter-bound GTFs.

## Author summary

Six general transcription factors (GTFs) assemble at promoters of protein-coding genes to enable recruitment of RNA polymerase II (RNAPII) and facilitate transcription initiation, but little is known about how they are regulated once promoter-bound. Here, we demonstrate that, in budding yeast, some components of GTFs are post-translationally modified by the SUMO peptide specifically when they are assembled at promoters. We determined that the large subunit of TFIIF, Tgf1, is the major target of sumoylation among GTFs and that increasing Tfg1 sumoylation reduces the interaction of TFIIF with RNAPII. Consistent with this, we found that increasing levels of SUMO at promoters of some protein-coding genes, by permanently attaching SUMO to Tfg1, resulted in reduced RNAPII levels associated with those genes. On the other hand, reducing promoter-associated sumoylation, by mutating SUMO-modified residues on Tfg1, also reduced RNAPII occupancy levels. Explaining these apparently contradictory findings, we propose that dynamic sumoylation of promoter-bound GTFs, not merely the presence or absence of SUMO, is important for facilitating rearrangements of promoter-bound GTF components that enhance transcription. Together, our data reveal a novel level of regulating the basal transcription machinery through SUMO modification at promoters of protein-coding genes.

## Introduction

Sumoylation is a conserved eukaryotic post-translational modification (PTM) that primarily affects nuclear proteins. It involves covalent conjugation with the SUMO peptide and has varied effects on its targets, including altered activity, localization, stability, and association with chromatin, which are often mediated by altering protein-protein interactions or through interplay with other PTMs [1–6]. Unlike the related process of ubiquitination, sumoylation in mammals and yeast involves a single E2 conjugating enzyme, Ubc9, and modifies specific Lys residues that are usually part of a SUMO consensus motif and lie within flexible, disordered regions of target proteins [6]. The level of sumoylation of individual substrates is typically low, which is partly due to the constitutive action of desumoylation enzymes, or SUMO proteases, including the SENP family in mammals, and Ulp1 and Ulp2 in budding yeast [7].

Transcription-related proteins are among the most frequently identified SUMO targets across species, implying that transcription is highly regulated by sumoylation. Such targets include about a third of the ~580 sumoylated proteins in budding yeast identified to date through proteomics analyses, and at least a quarter of the nearly 6400 sumoylated proteins identified in cultured human cells grown in unstressed conditions [6,8–10]. Among these are numerous sequence-specific transcription factors (SSTFs), > 200 of which have been studied individually to examine the effects of their sumoylation [3]. For example, sumoylation of Elk-1 was shown to facilitate recruitment of the histone deacetylase corepressor complex HDAC-2, thereby switching from a transcriptionally active role to repressing target genes, whereas for delta-lactoferrin, sumoylation competes with acetylation and ubiquitination at different Lys residues to repress transcription or block degradation of the SSTF, respectively [11,12]. A more general role for sumoylation of SSTFs, however, may lie in regulating their association with chromatin. In studies in which chromatin immunoprecipitation (ChIP) analysis was performed, sumoylation-impairing mutations were frequently observed to cause increased chromatin occupancy levels of diverse SSTFs, such as yeast Gcn4 and human FOXA1 [3,13–15]. Moreover, in genome-wide ChIP (ChIP-seq) analyses performed with Sko1 in budding yeast, human MITF, and the human androgen and glucocorticoid receptors, sumoylation-impairing mutations led to a dramatic increase in the number of genomic binding sites at loci not normally bound by these SSTFs [16–21]. These findings point to general and conserved roles for sumoylation of SSTFs in promoting dissociation from nonspecific sites genome-wide, while limiting their association with authentic binding sites [3,22].

In addition to SSTFs, multiple components of the general transcription machinery, including subunits of the general transcription factors (GTFs) and RNA polymerases (RNAPs) I, II, and III, have been identified as SUMO targets, but the effects of SUMO modification of these proteins have been examined in only a few cases [2,6,9,23]. Under normal growth conditions, sumoylation of the yeast RNAPIII subunit Rpc82 promotes assembly and recruitment of RNAPIII to tRNA-encoding genes, whereas under conditions of stress or nutrient depletion, under which tRNA transcription is reduced, multiple RNAPIII subunits become unsumoylated [23,24]. Although these data strongly link RNAPIII sumoylation with a positive effect on its transcriptional activity, using genetic analysis, another study found that impairing sumoylation could at least partly rescue the growth defects associated with mutant forms of RNAPIII subunits, suggesting that in some conditions, sumoylation can repress RNAPIII activity [25]. Also in yeast, the elongation-associated form of RNAPII was found to become sumoylated on its large subunit, Rpb1, in response to UV irradiation or impairment of transcription, implicating the modification in the resolution of transcriptionally stalled RNAPII complexes at DNA lesions [26,27]. In human cells, both TBP and multiple TAF subunits of TFIID are known SUMO targets, and in in vitro assays, sumoylation of hsTAF5 was found to inhibit binding of this GTF to an immobilized DNA template, suggesting that TFIID sumoylation might be involved in regulating the assembly of the complex with promoters [6,9,28,29]. Components of yeast RNAPII-associated GTFs, including multiple subunits of TFIIA, TFIID, TFIIE, and TFIIF, have been frequently identified in mass spectrometry analyses of the SUMO proteome [8,10,30–33]. However, how SUMO regulates their individual functions, or whether it controls the transcriptional preinitiation complex (PIC) assembled on active gene promoters, has not been examined.

When exploring the effects of SUMO on transcription, a number of early studies pointed to repressive roles for sumoylation of transcription-related proteins [34–36]. However, in 2010 we reported the first SUMO ChIP analysis, in which sumoylated proteins were detected on promoter regions of a number of transcriptionally active genes in yeast, but not at the repressed/silenced genes that were examined [37]. The association of SUMO with promoters of active genes was subsequently validated genome-wide in a series of ChIP-seq studies. In human cells, SUMO isoforms are widely distributed across the genome but are primarily enriched at promoters, specifically at transcriptional start sites (TSSs), of many, but not all, highly-expressed genes [38,39]. Among the gene types most likely to show promoter-associated SUMO are those involved in translation, including ribosomal protein genes (RPGs), and genes encoding histones, rRNAs, and tRNAs, but the identity of the sumoylated factor(s) at these genes has not been determined [39]. In a ChIP-seq analysis performed in yeast, FLAG epitope-tagged SUMO was detected with high confidence at 423 loci across the genome and these were associated almost exclusively with RPGs and tRNA genes [40]. The investigators demonstrated that SUMO peaks associated with the RPGs, which are situated upstream of TSSs, derive specifically from sumoylated Rap1, a multifunctional SSTF known to bind to many gene types, including RPGs [41]. Furthermore, Rap1 sumoylation was shown to be required for transcription of RPGs, likely by facilitating an interaction with TFIID and enhancing its recruitment to target gene promoters [40]. Whereas this study convincingly posits that Rap1 is responsible for SUMO peaks associated with RPGs, and previous work from the same group implies that SUMO detected at tRNA genes derive from sumoylated RNAPIII subunits, the nature of sumoylation associated with protein-coding genes that are not RPGs (referred to hereafter as non-RPGs) remains unexplored [23].

Here we demonstrate that GTFs are sumoylated at promoters of a subset of non-RPGs in yeast, with TFIIF specifically showing the highest level of sumoylation. We performed SUMO ChIP-seq in a genetically unmodified lab yeast strain and identified 147 robust SUMO peaks associated with non-RPGs, most of which align perfectly with the binding sites of GTFs but not at the binding sites of other known promoter-associated factors. Intriguingly, non-RPGs that show high levels of transcriptional activity and high TFIIF occupancy are more likely to harbour detectable sumoylation and, correspondingly, highly-active SUMO peak-containing non-RPGs show significantly reduced RNAPII occupancy when cellular sumoylation levels are impaired. This implies a positive role for promoter-associated SUMO in enhancing transcription, but it may also reflect higher detectability of SUMO-modified GTFs at sites with high levels of GTFs, in which case sumoylation at non-RPGs may be more widespread than we have observed. Furthermore, we find that Tfg1, the largest subunit of TFIIF, is the most sumoylated GTF component, and identify its sumoylation site to be Lys residues 60/61, which are situated in a disordered, accessible region of TFIIF when it is associated with the PIC [42]. Finally, we provide evidence that dynamic sumoylation of promoter-bound TFIIF, and likely other GTF components, acts to facilitate transcription and increase RNAPII occupancy levels.

## Results

### Sumoylated proteins stably associate with promoters of a subset of non-RPG protein coding genes

**Fig 1:**
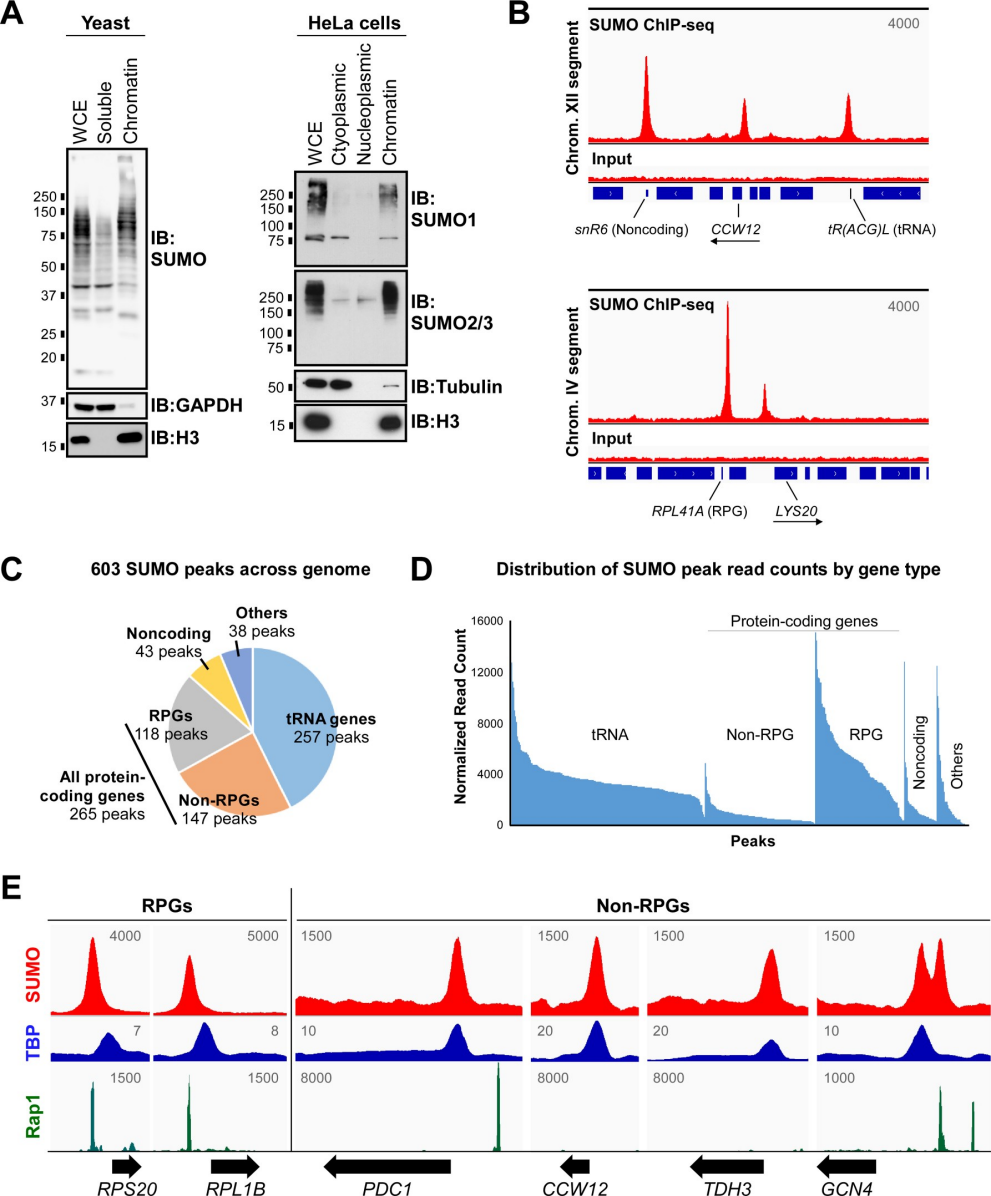
Sumoylated proteins associate stably with chromatin, including at many non-RPGs. **(A)** Most sumoylated proteins are associated with chromatin. Fractionation experiments were carried out in normally growing, unmodified yeast and human HeLa cells, then whole cell extracts (WCE) and indicated fractions were analyzed by immunoblot (IB) with indicated antibodies, including antibodies for the yeast Smt3 peptide (“SUMO”), human SUMO1 or SUMO2/3 isoforms, and histone H3. **(B)** Independent duplicate SUMO ChIP-seq experiments were performed in yeast, and sample alignments are shown from Replicate 1, using the Integrative Genomics Viewer (IGV) genomic alignment tool, with reads from the SUMO IP and corresponding inputs over selected short segments of Chromosomes XII and IV. Values correspond to maximum data range (read numbers) for the view shown and blue bars along the bottom represent gene positions, including for two non-RPGs, *CCW12* and *LYS20*, whose ORF orientations are indicated with arrows. See S9 Table for a list of yeast genes associated with the 603 SUMO peaks. **(C)** Pie chart showing fractions of the 603 SUMO ChIP-seq peak set associated with different gene types. Non-RPG refers to protein-coding genes that are not ribosomal protein genes (RPGs). See S2 Table for detailed description of peak classifications. **(D**) Distribution of read counts for the 603 SUMO ChIP-seq peaks, separated by gene type, then ranked by normalized read counts. Normalized read counts were determined using DiffBind tool with two independent ChIP-seq replicates. **(E)** Sample SUMO peak alignments from Replicate 1 are shown for two RPGs and four non-RPGs. Peak alignments for TBP and Rap1 are also shown, using published ChIP-seq and ChIP-exo datasets, respectively, for comparison with SUMO peak positions (NCBI GEO database accession numbers GSM2870615 and GSE93662, respectively). Values refer to maximum data range (read numbers) for the view shown, and unnormalized alignments were generated using IGV.

Proteomics analysis of sumoylated proteins in yeast, mammals, and other species revealed that numerous proteins that associate with chromatin are SUMO targets [3,9,43,44]. To determine to what degree SUMO modifies proteins specifically when they are associated with chromatin, we carried out fractionation experiments in both budding yeast and human HeLa cells. As shown in Fig 1A, the vast majority of sumoylated proteins in both organisms are chromatin-bound, supporting the notion that SUMO functions primarily in regulating processes associated with chromatin, such as gene regulation. To examine specifically where sumoylated proteins are stably bound to the yeast genome, we carried out a ChIP-seq experiment using a genetically unmodified, normally growing strain (W303a) and an antibody that is specific for yeast SUMO [45]. Stringent analysis of two independent SUMO ChIP-seq replicates identified a common set of 603 robust SUMO peaks across the yeast genome (S1A Fig and S2, S4 and S9 Tables). Examples of peaks associated with different gene types are shown in Fig 1B. According to this analysis, the bulk of sumoylated proteins that are stably associated with chromatin (i.e. readily detected by SUMO ChIP) are situated at genes encoding tRNAs and at RPGs, which is highly consistent with the findings of a previous study in which the genome occupancy of FLAG epitope-tagged yeast SUMO was determined by FLAG ChIP-seq (Fig 1C and 1D; [40]). However, in contrast to the previous study, we also identified a significant number (147) of high-stringency SUMO peaks associated with non-RPGs (Figs 1C, 1D and S1B). This implies that the expression of a subset of this class of genes is regulated by chromatin-associated sumoylation in normally growing yeast.

In the study by Chymkowitch et al, SUMO peaks associated with RPGs were found to derive from the sumoylated transcription factor Rap1 [40]. Using published ChIP-seq datasets, we compared the genomic positions of SUMO peaks from our study with the binding sites of Rap1 and the TATA-box binding protein, TBP. Supporting the previous study, virtually all identified SUMO peaks at RPGs aligned perfectly with Rap1, which binds further upstream from transcriptional start sites (TSSs) than TBP does (e.g. RPGs in Fig 1E). Although Rap1 is known to also bind and regulate many non-RPGs [41], only 14 of the 147 SUMO peaks at non-RPGs aligned with known Rap1 binding sites. A small number of non-RPGs are associated with two distinct but adjacent SUMO peaks, only one of which aligned with Rap1 (e.g. *GCN4* in Fig 1E), whereas most known Rap1 binding sites that are situated at non-RPGs did not align with SUMO peaks (e.g. at *PDC1* in Fig 1E). Intriguingly, however, the remaining 133 SUMO peaks at non-RPGs, including the non-Rap1 peak at *GCN4*, aligned perfectly with TBP (Fig 1E). As TBP binds near the transcriptional start site (TSS) as part of a complex of proteins that form the PIC, our analysis indicates that SUMO regulates one or more proteins bound to the core promoters of a subset of non-RPGs.

### Stable SUMO peaks are primarily detected at promoters of highly transcribed non-RPGs

**Fig 2:**
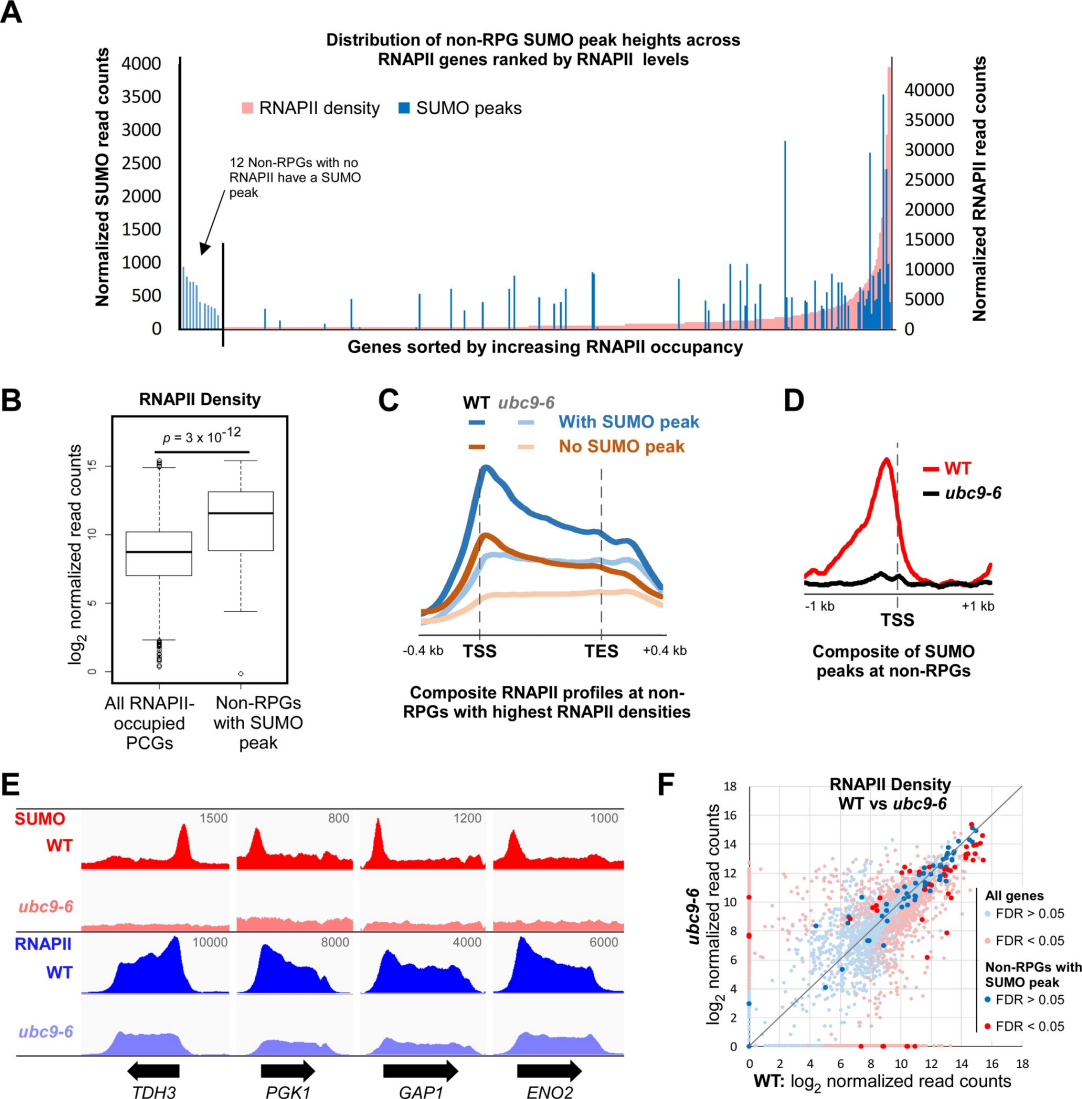
Many highly transcribed non-RPGs have a promoter-associated SUMO peak. **(A)** Comparison of SUMO peak-containing genes by transcription level. The 111 SUMO peaks (blue) associated with single non-RPGs were plotted, by normalized read count, over the ranked distribution of RNAPII densities (pink) of all protein-coding genes (PCGs), which are approximations of transcription levels and were determined by duplicate independent RNAPII ChIP-seq analyses performed in the same strain and conditions as the SUMO ChIP-seq. Inset shows 12 SUMO peaks of the set of 111 that are associated with genes that have no detected RNAPII density. See S11 Table for list of RNAPII densities by gene. **(B)** Box plot comparing RNAPII densities for all RNAPII-occupied genes and for the 111 non-RPGs with unique SUMO peaks. *P*-value of Student’s *t*-test analysis is shown. **(C)** Composite plots of RNAPII ChIP-seq profiles in WT or *ubc9-6* cells at non-RPGs with the highest RNAPII densities (top 10%) that either contain or lack promoter-associated SUMO peaks, as indicated. Plots were generated using the ComputeMatrix tool (from deepTools 3.3.0). *TSS*, transcription start site; *TES*, transcription end site. **(D)** Composite plots of SUMO peaks associated with single non-RPGs in WT or *ubc9-6* cells, shown relative to nearest TSS, generated using ComputeMatrix. **(E)** Unnormalized ChIP-seq peak alignments for SUMO and RNAPII in WT and *ubc9-6* cells at four selected genes with normally high RNAPII densities (i.e. in WT cells). Values refer to maximum data range (read numbers) for the view shown per gene for both WT and corresponding *ubc9-6* alignments. **(F)** Plot of RNAPII densities of all protein-coding genes in WT versus *ubc9-6* cells, with densities at the 111 non-RPGs associated with unique SUMO peaks highlighted. See S11 Table for list of RNAPII densities in WT and *ubc9-6* strains by gene.

Because some of the 133 non-RPG SUMO peaks that align with TBP are situated at overlapping promoters of two closely spaced divergent genes, 155 non-RPG genes in total appear to be regulated by sumoylation of promoter-bound proteins (S9 Table). To determine whether these genes are functionally related, we carried out gene ontology (GO) term analysis, which revealed that almost a quarter of these genes encode proteins that are involved in the biosynthesis of small molecules, including nucleotides, suggesting that this type of genes may be particularly subject to regulation by SUMO (S10 Table). Next, we wished to determine whether the presence of SUMO peaks at non-RPGs correlates with their transcriptional activity. Particularly in yeast, where transcriptional pausing is not prevalent, levels of RNAPII associated with genes can be a reasonable measure of transcriptional activity [46]. As such, we determined the density of RNAPII across the open reading frames (ORFs) of all protein coding genes (PGCs) by ChIP-seq in the same strain and conditions used for the SUMO ChIP-seq and using an antibody that recognizes the largest RNAPII subunit (8WG16 antibody). Fig 2A shows the distribution of the 111 non-RPGs that have a unique SUMO peak (i.e. not shared between two divergent genes) overlaid on the ranked distribution of RNAPII levels across all ORFs that contain detectable RNAPII levels. Although some SUMO-peak containing non-RPGs show little or no RNAPII occupancy, a significant number is clustered at the end of the range with high levels of RNAPII. Indeed, over half of non-RPGs with a SUMO peak (51%) are among the top 10% most transcribed genes, as measured by RNAPII density, and as a whole, the 111 non-RPGs have a significantly higher than average level of RNAPII (Fig 2A and 2B). Even among the top 10% most transcribed non-RPGs, those with a SUMO peak on average show distinctly higher RNAPII densities than those lacking a peak (Fig 2C). Although, the intensities of the SUMO peaks themselves (“peak levels”) do not correlate with RNAPII densities (S1C Fig), this analysis indicates that promoter-associated sumoylation is a feature of many highly transcribed genes.

The association of SUMO with highly transcribed genes suggests that promoter-associated sumoylation may function to elevate transcription levels. To explore this possibility, we examined the effect of reduced sumoylation on RNAPII density through RNAPII ChIP-seq in a yeast strain, *ubc9-6*, which harbors a mutation in Ubc9 that reduces its activity and virtually abolishes SUMO peaks at promoters of non-RPGs (Figs 2D, 2E, S1D, S1E and S1F). Of the 111 non-RPGs with a unique SUMO peak, 44 showed a significant change in RNAPII density in the *ubc9-6* strain, about two-thirds of which showed reduced RNAPII (Fig 2E and 2F). However, the *ubc9-6* mutation affected RNAPII levels at hundreds of genes overall, including those that do not have a detectable SUMO peak (Fig 2F and S11 Table). Notably, among highly transcribed genes, reduced global sumoylation had a similar effect in reducing RNAPII levels on genes that contain SUMO peaks and on those that lack detectable SUMO peaks (Fig 2C). This indicates that reduced cellular sumoylation in the *ubc9-6* strain affects transcription generally, not only at non-RPGs that normally have SUMO peaks at their promoters. A possible explanation, which we explore below, is that promoter-associated sumoylation at non-RPGs is far more widespread than we are able to detect, such that impairing sumoylation affects transcription of a larger number of genes than we initially expected. Nonetheless, whereas only ~13% of all protein-coding genes show reduced RNAPII levels in *ubc9-6*, this number rises to ~26% for SUMO peak-containing non-RPGs, suggesting that detectable levels of promoter-associated sumoylation can have a positive effect on transcription. Indeed, the analysis shown in Fig 2F, in which SUMO-containing non-RPGs are overlaid on all genes, also highlights that many of the most highly transcribed genes have a detectable SUMO peak. Notably, 24 of the 25 SUMO peak-containing non-RPGs that are among the top 1% of genes with highest RNAPII densities showed diminished transcription when sumoylation was reduced.

### SUMO at promoters of non-RPGs derives from sumoylated GTF components

Towards identifying the sumoylated protein(s) associated with promoters of non-RPGs, we analyzed DNA sequences encompassing the SUMO peaks. First, MEME motif analysis was used to identify ungapped recurring patterns [47]. As a control, we first applied this tool to the set of 118 RPGs with a SUMO peak and it successfully identified a motif, present in 113 of the sequences, that matches the Rap1 consensus motif (S2A Fig). This further supports the previous study that showed that SUMO peaks at RPGs derive from Rap1 sumoylation [40]. When applied to the 133 non-RPGs that lack a known Rap1 binding site, MEME analysis identified only a somewhat indistinct AG-rich sequence in 38 of the sequences (S2A Fig). We then applied the DREME motif analysis tool to identify relatively enriched short motifs (up to 8 nt in length) in sequences surrounding SUMO peak summits. The most significant motif identified by this analysis (RTATAWA), present in 94 of the 133 non-RPG SUMO peak sequences, shows strong similarity to the TATA box element (consensus sequence TATAWAWR; S2B Fig; [41]). This is highly consistent with our finding that these SUMO peaks align with the position of TBP, which binds the TATA element or similar sequences at most promoters [41], and supports the notion that SUMO peaks at non-RPGs derive from sumoylated proteins associated with the core promoter of these genes.

**Fig 3:**
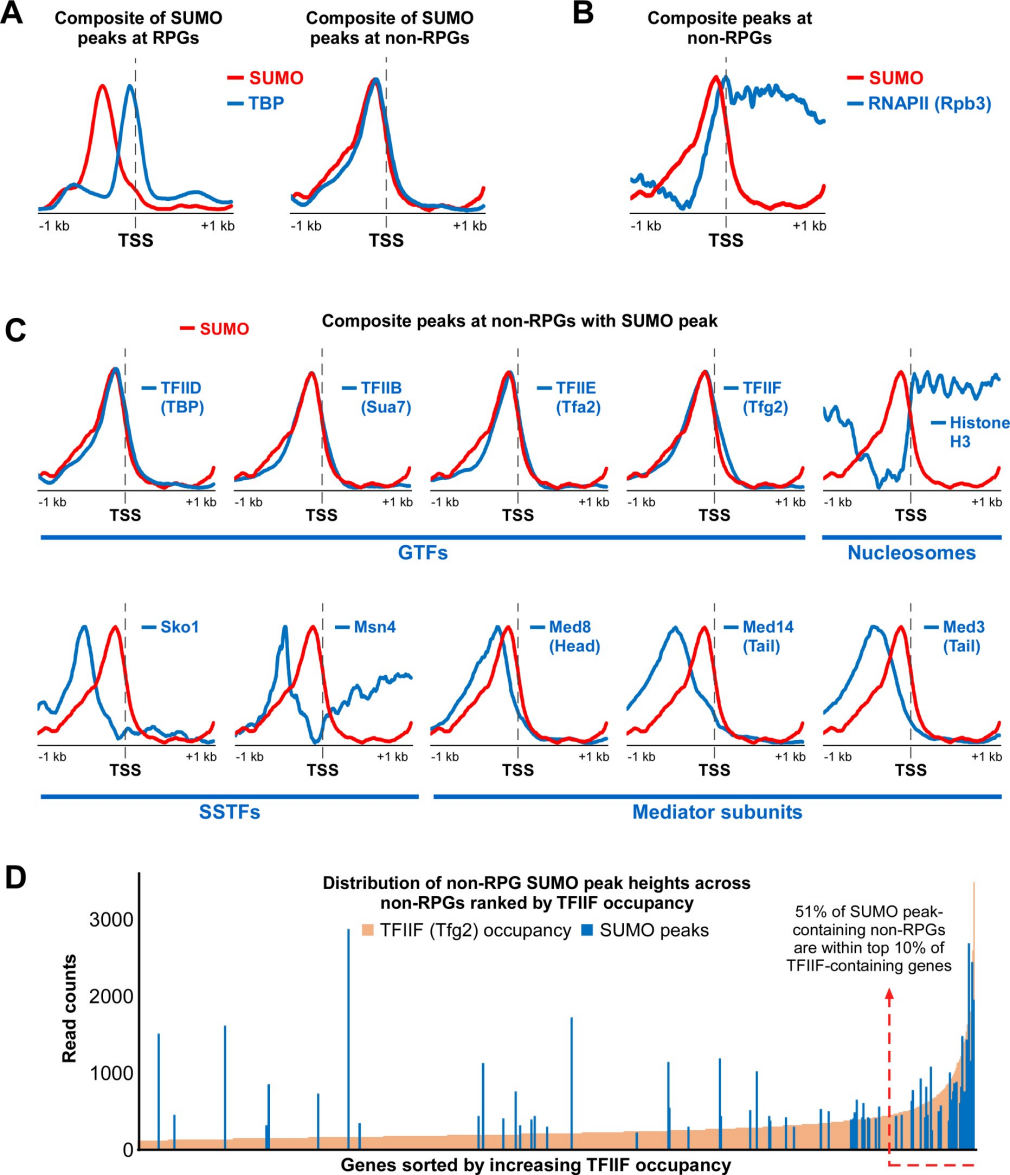
SUMO peaks at promoters of non-PRGs align specifically with GTF components. **(A)** Comparison of SUMO and TBP composite plots at SUMO peak-containing RPGs (left) and non-RPGs (right), shown relative to the TSS position. SUMO plots were generated with data from Replicate 1 of our SUMO ChIP-seq analysis, whereas TBP data were obtained from NCBI GEO database (accession number GSM2870615). **(B)** Comparison of SUMO and RNAPII (subunit Rpb3) composite plots at SUMO peak-containing non-RPGs. Rpb3 data were obtained from the GEO database (accession number GSM3629815). **(C)** Composite plots for GTF subunits, histone H3, sequence-specific transcription factors (SSTFs), and Mediator subunits were compared with SUMO composite plots at SUMO peak-containing non-RPGs. All datasets, except for SUMO (as described in *A*), were obtained from the GEO database, with the following accession numbers: Sua7 (TFIIB subunit): GSM4319112; Tfa2 (TFIIE subunit): GSM4319116; Tfg2 (TFIIF subunit): GSM4319120; H3: GSM2561057; Sko1: GSM3335975; Msn4: GSM1859030; Med8 (Mediator head subunit): GSM3189528; Med14 (Mediator tail subunit): GSM3189529; and Med3 (Mediator tail subunit): GSM3189530. **(D)** Distribution of SUMO peak-containing non-RPGs by TFIIF occupancy level. The 111 SUMO peaks (blue) associated with single non-RPGs were plotted, by normalized read count, over the ranked distribution of TFIIF (subunit Tfg2) occupancy levels (ChIP-seq counts per million) over all Tfg2-containing non-RPGs. Tfg2 occupancy levels derived from GEO database (accession GSM4319120).

Next, we generated summary distribution plots (“meta-gene” composites) of all SUMO peaks relative to their nearest TSS and compared them to similar plots of proteins known to bind at or near promoters, generated using published ChIP-seq datasets. As shown in Fig 3A, performing this analysis with SUMO peaks associated with RPGs shows a composite plot that peaks ~400 bp upstream of the TSS, consistent with the position of Rap1 binding [41], whereas the composite peak for TBP at these genes is situated immediately upstream of the TSS, as expected. Analysis of SUMO peaks associated with non-RPGs, however, shows a composite SUMO plot that almost perfectly overlaps the TBP composite peak at these genes, which is particularly notable considering that the SUMO and TBP ChIP-seq analyses were performed years apart in different labs (Fig 3A). Although some RNAPII subunits have been identified as SUMO targets (see S2C Fig; [8,10,30,31]), the composite SUMO plot at non-RPGs did not match the composite plot of the RNAPII subunit Rpb3 (Fig 3B). Similarly, composite plots for histone H3, components of the Mediator, and two sequence specific transcription factors (SSTFs), Sko1 and Msn4, did not match the plot for SUMO at non-RPGs (Fig 3C). In contrast, as with TBP, plots for components of GTFs, including subunits of TFIIB, TFIIE, and TFIIF, all overlapped well with the non-RPG SUMO composite plot (Fig 3C). The composite plots for GTF components are largely indistinguishable from each other, which likely reflects their close proximity when assembled at core promoters and the limited resolution of conventional ChIP (e.g. [48]). In any case, this analysis strongly suggests that SUMO peaks at non-RPGs derive specifically from promoter-bound GTF components and not from other proximally bound proteins.

To explore this further, we compared the genome distribution of non-RPG SUMO peaks with the distribution of a GTF component, TFIIF, as determined by analysis of a previously reported Tfg2 ChIP-seq dataset [49]. If SUMO peaks at promoters of non-RPGs derive from sumoylated GTFs, then we might expect SUMO to be more readily detected on genes that have high levels of GTF components. Fig 3D shows the distribution of TFIIF-containing non-RPGs, ranked by increasing Tfg2 occupancy levels, overlaid with SUMO peak levels at these genes. Indeed, although there are some notable exceptions, the majority of non-RPG SUMO peaks are found on genes with high TFIIF levels (top 10%). To examine this in a manner that does not depend on potentially biased processing steps like assigning peaks to specific genes, we compared the distribution of raw read counts at TFIIF peaks genome-wide (unassigned to any genes) with the raw read counts of SUMO at each of these peak positions. As shown in S2D Fig, genomic sites showing the highest raw read counts of TFIIF also show the highest levels of SUMO. This further supports the idea that SUMO at non-RPG promoters derives from GTF components but, importantly, it also suggests that we were able to detect GTF-associated SUMO effectively only at loci with the highest levels of GTFs. In other words, it is possible that GTF sumoylation occurs widely at promoters of non-RPGs, but our detection threshold only allows us to observe it where GTFs are most stably bound (i.e. have high ChIP occupancy levels).

### Chromatin-associated TFIIF is sumoylated at Lys 60/61 of its Tfg1 subunit

**Fig 4:**
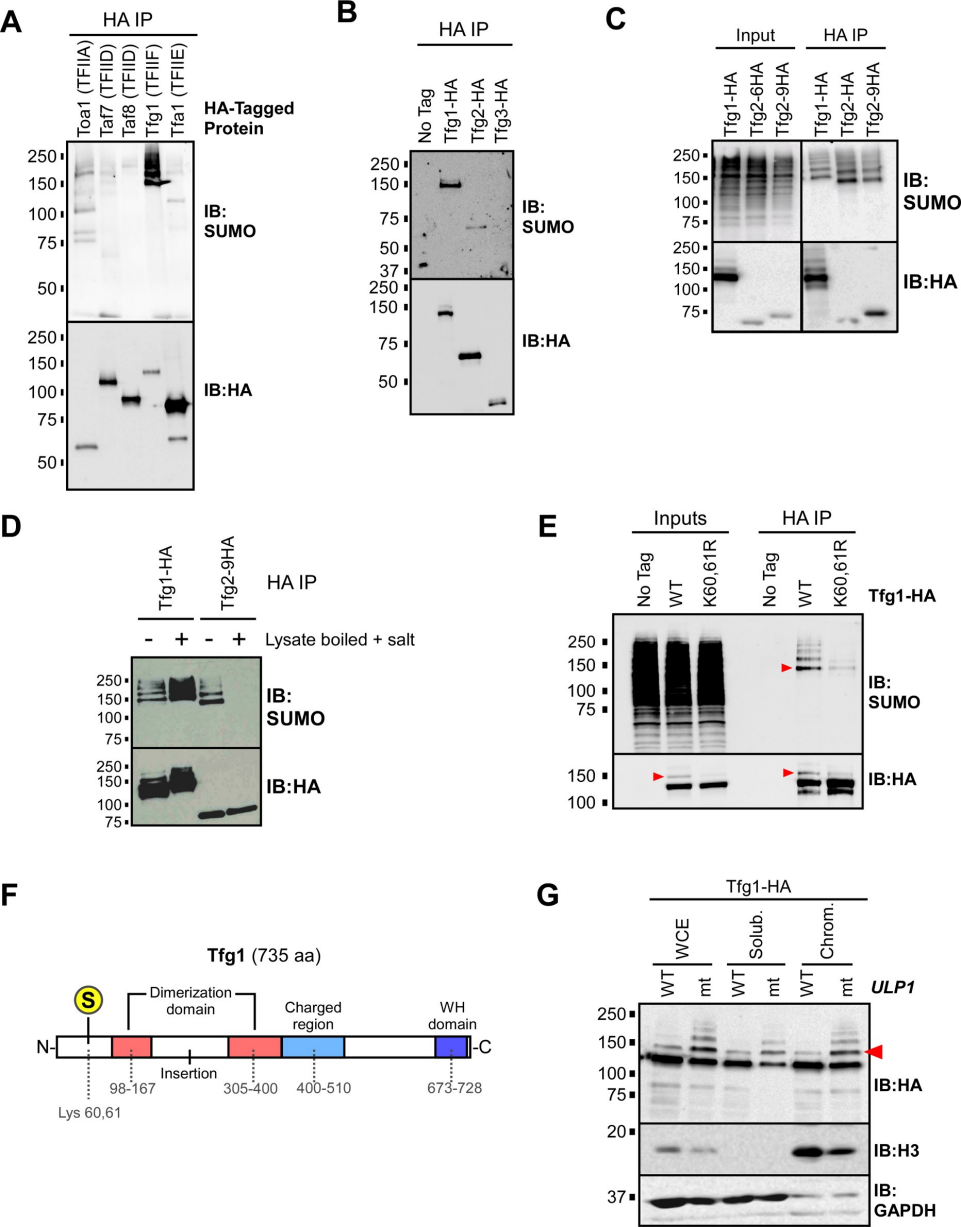
The large subunit of TFIIF, Tfg1, is sumoylated at Lys 60/61. **(A)** Yeast strains were generated that each express a 6xHA C-terminal epitope tag on a different GTF subunit, including Toa1 (TFIIA), Taf7 (TFIID), Taf8 (TFIID), Tfg1 (TFIIF), or Tfa1 (TFIIE). Cultures of the strains were used to prepare lysates, under non-denaturing conditions, that were then used in HA IP experiments, followed by SUMO and HA immunoblots (IBs). **(B)** Strains expressing HA-tagged forms of TFIIF subunits Tfg1, Tfg2, or Tfg3 (also known as TAF14), were used for HA-IP experiments followed by SUMO and HA immunoblot analysis. “No tag” refers to the parental strain (W303a) that expresses no HA-tagged proteins. **(C)** Strains expressing Tfg1-HA, Tfg2-HA, or Tfg2-9HA (with a 9xHA tag instead of the usual 6xHA tag) were used in an HA IP analysis performed with lysates generated under non-denaturing conditions, followed by HA and SUMO immunoblots. The pattern detected in the SUMO blot of the Tfg2 IPs likely corresponds to sumoylated Tfg1 which coIPs with both forms of Tfg2. In the Tfg1-HA IP lane, the sumoylated species migrate slower because sumoylated Tfg1-HA includes the 6xHA tag, whereas Tfg1 (and its sumoylated forms) is untagged in the Tfg2-6HA and Tfg2-9HA strains. **(D)** Lysates were prepared from Tfg1-HA and Tfg2-9HA strains, which were then either treated (+) or not treated (-) by boiling for 5 min and adjusting the NaCl concentration to 0.5 M to promote the disruption of protein complexes prior to IP. Lysates were then used for HA IP analysis followed by SUMO and HA immunoblots. The pattern seen in the SUMO blot of the Tfg2-9HA IP disappears in the treated sample, implying that these sumoylated species are indeed derived from coIPed, sumoylated Tfg1. Note that treatment appears to elevate Tfg1 sumoylation levels (compare first two lanes in SUMO blot), likely because it inactivates naturally occurring SUMO proteases present in the lysate. **(E)** A mutant strain was generated that expresses Tfg1-HA with Arg substitutions at Lys 60 and 61 (K60,61R). This strain, along the WT Tfg1-HA-expressing strain, were used in HA IP-immunoblot experiments, and SUMO and HA immunoblots are shown. To disrupt protein-protein interactions, NaCl concentration was increased to 0.5 M and lysates were then boiled for 5 min, then cooled on ice prior to IP. Inputs represent approximately 5% of the material used for IP. **(F)** Diagram of Tfg1 domain structure, based on [64]. The major SUMO acceptor site is indicated with an encircled *S* at Lys 60/61. **(G)** Sumoylated Tfg1 associates with chromatin. A strain harboring a point mutation (I615N) in *ULP1* (mt), and an isogenic wild-type strain (WT), were engineered to express Tfg1-HA, then the strains were used for chromatin fractionation analysis. Whole-cell extract (WCE), soluble, and chromatin fractions were analyzed by HA, histone H3, and GAPDH immunoblots. Red arrowheads indicate the position of mono-sumoylated Tfg1 in HA and SUMO immunoblots throughout.

Of the six GTFs that form pre-initiation complexes at core promoters, subunits of four have been identified as putative SUMO targets through several proteomics studies in yeast (TFIIA, TFIID, TFIIE, and TFIIF; S2C Fig). To determine which GTFs are sumoylated in the yeast strain and conditions used in our ChIP-seq studies, we generated derivative strains that each express a 6X-HA epitope-tagged version of select GTF subunits, from their natural genomic loci. The proteins were immunoprecipitated (IPed) from cell lysates prepared under nondenaturing conditions and examined by SUMO and HA immunoblots. By this method, bands appearing on SUMO immunoblots can derive either from sumoylated versions of the HA-tagged GTF subunits themselves or from tightly associated co-IPed proteins that are sumoylated. As shown in Fig 4A, sumoylated species were detected in the IPs for subunits of all the four GTFs. In dramatic contrast to the other GTFs, however, the IP for the large subunit of TFIIF (Tfg1), showed an intense signal on the SUMO blot, at the position expected for sumoylated Tfg1, and in a ladder pattern that is characteristic of proteins that are multi- and/or polysumoylated. Supporting the observation that Tfg1 is highly sumoylated, it is the only GTF subunit that was identified in all six SUMO proteomics studies listed in S2C Fig [8,10,30–33].

Yeast TFIIF consists of two major subunits, Tfg1 and Tfg2, and a third, less tightly-associated subunit that is also a component of TFIID, named Tfg3 or TAF14 [50]. To confirm that Tfg1 is the major sumoylated component of TFIIF, we prepared lysates from strains expressing HA-tagged Tfg1, Tfg2, or Tfg3 under conditions that disrupt protein interactions, followed by HA IP and HA and SUMO immunoblots. We found that Tfg2 shows a modest level of sumoylation, but the major sumoylated species in TFIIF is indeed Tfg1 (Fig 4B). Notably, SUMO immunoblots of Tfg2-HA IPs show coIPed sumoylated species that are consistent with the size and pattern of sumoylated Tfg1 only if lysates were prepared in conditions that retain protein-protein interactions (Fig 4C and 4D). This confirms that our IP-immunoblot analyses performed with non-denatured lysates can readily detect sumoylated co-IPed proteins. Since Tfg1 is by far the most sumoylated species detected in the non-denaturing IP-immunoblot analysis shown in Fig 4A, these data imply that Tfg1 is the most highly sumoylated GTF component in normally growing yeast.

Next, we sought to identify the sumoylated residues on Tfg1. Computational sequence analysis using SUMO site prediction algorithms identified multiple (five to ten) potential SUMO sites on Tfg1, but only four Tfg1 peptide fragments were detected in a proteomics analysis that mapped SUMO acceptor sites in budding yeast, corresponding to Lys residues at positions 60, 91, 574, and 658 [10]. To determine which residues are responsible for the relatively high level of Tfg1 sumoylation, we generated strains expressing Tfg1-HA with Lys-to-Arg mutations at different putative modification sites, then determined whether the mutations reduce Tfg1 sumoylation levels. Using the HA IP-immunoblot method described above, we found that mutation of Lys 91, 658, or a computationally-predicted SUMO acceptor residue, Lys 733, did not significantly affect Tfg1 sumoylation levels (S3A Fig). To examine Lys 60, which is adjacent to another Lys residue (Lys 61), we generated a strain in which both Lys residues 60 and 61 are replaced with Arg to ensure that sumoylation is not possible at this site. As shown in Fig 4E, the K60,61R mutation dramatically reduces, but does not abolish, sumoylation of Tfg1, indicating that K60,61 is its major SUMO acceptor site and that there are one or more minor additional acceptor sites on the protein. The major sumoylation site on Tfg1, therefore, lies within its N-terminal region, upstream of the Tfg1-Tfg2 dimerization domain (Fig 4F). Strains expressing the Tfg1 K60,61R mutation grew normally under standard and stress conditions, indicating that blocking sumoylation at these residues is not sufficient to affect cell growth or viability (S3B, S3C and S3D Fig), but we discuss the potential significance of SUMO modification at this site below (see Discussion).

HA immunoblot analysis of Tfg1-HA derived from lysates prepared with *N*-ethylmaleimide (NEM), which inhibits SUMO proteases, shows a higher molecular weight species that is greatly reduced in the K60,61R strain and co-migrates with the major sumoylated form of Tfg1-HA on SUMO blots, implying that mono-sumoylated Tfg1-HA can be detected on HA immunoblots (red arrowheads in Fig 4E). This is further supported by the fact that this species becomes relatively more intense in a strain harboring a partially defective form of the Ulp1 SUMO protease (*ulp1-mt*; Figs 4G and S3F), and because it co-migrates with a SUMO-Tfg1 fusion polypeptide (see below). Taking advantage that both unmodified and mono-sumoylated Tfg1-HA can be detected on the same immunoblot, through densitometry of HA immunoblots, we determined that approximately 10% to 15% of Tfg1 molecules are mono-sumoylated in normally growing yeast. We also took advantage of this to determine whether sumoylated Tfg1 specifically is associated with chromatin by fractionation followed by HA immunoblot. To better detect sumoylated Tfg1, we performed the fractionation experiment in the *ulp1-mt* background strain, as well as in its isogenic WT *ULP1* parent. As shown in Fig 4G, both unmodified and sumoylated Tfg1 are detected in chromatin and soluble fractions. These data demonstrate that SUMO regulates properties of TFIIF while it is associated with chromatin.

### Elevated sumoylation reduces the interaction of TFIIF with RNAPII

**Fig 5:**
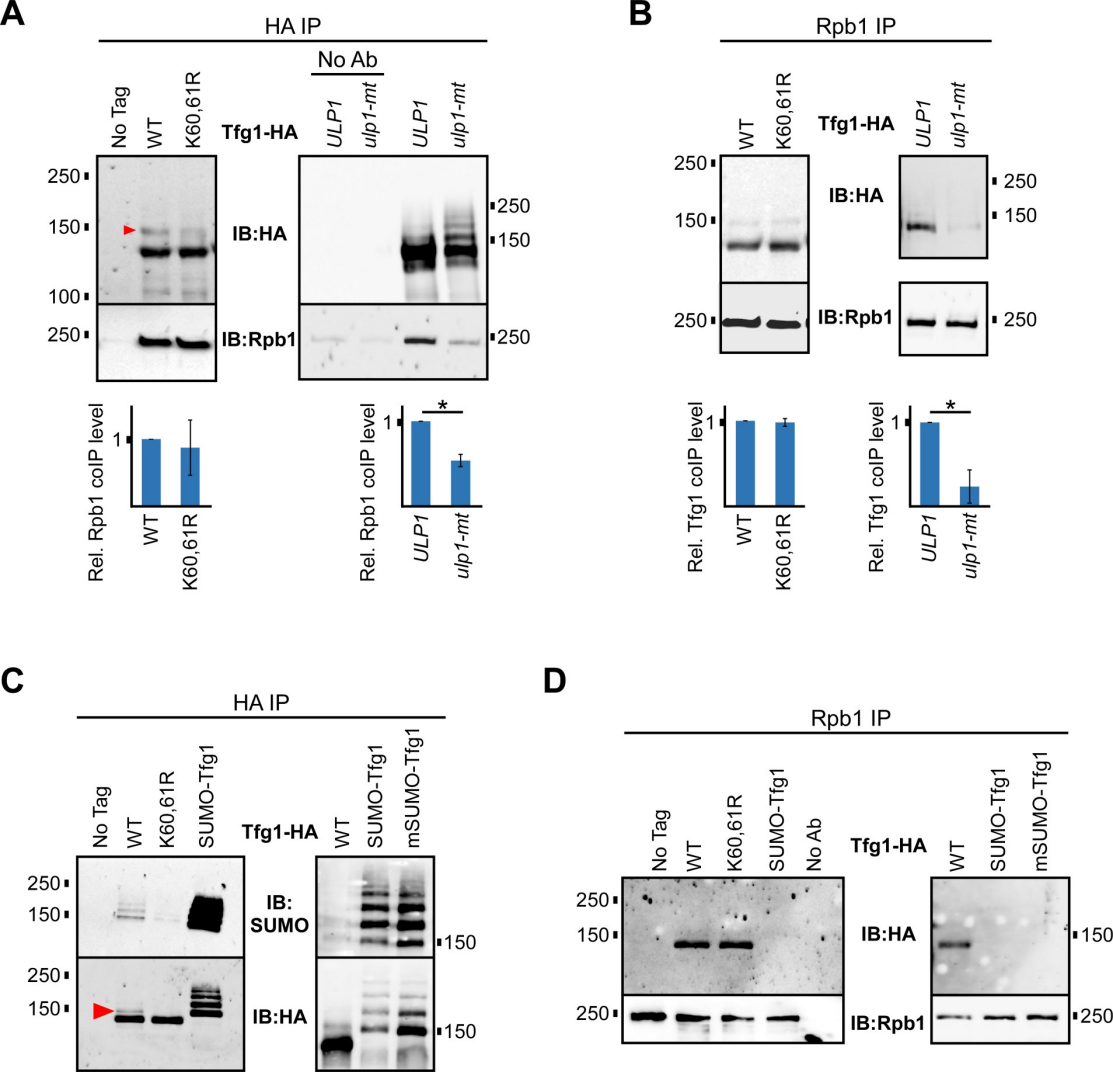
Elevated Tfg1 sumoylation reduces its interaction with RNAPII. **(A)** Tfg1-HA and Tfg1-K60,61R-HA strains, and strains expressing Tfg1-HA in the *ulp1-mt* (I615N) or *ULP1* parental backgrounds, were used for co-IP experiments. HA IPs, and no antibody (No Ab) controls, were analyzed by HA and Rpb1 (8WG16 antibody) immunoblots. Relative levels of co-IPed Rpb1, normalized to Tfg1-HA IP levels, were determined by densitometry and average values from at least three experiments are shown. Student’s *t*-tests were performed and paired values that are statistically different (*p* value < 0.05) are indicated with an asterisk. **(B)** Using the same strains described in *A*, Rpb1 IP was performed, followed by HA and Rpb1 immunoblots. Quantification of levels of co-IPed Tfg1-HA, normalized to IPed Rpb1 levels, was performed as in *A*. **(C)** Strains expressing Tfg1-HA with an N-terminal fusion to the yeast SUMO peptide (SUMO-Tfg1) or to a mutant form of the SUMO peptide in which all Lys residues are replaced with Ala (mSUMO-Tfg1), were analyzed alongside strains expressing WT and K60,61R forms of Tfg1-HA in a HA IPs, followed by SUMO and HA immunoblots. **(D)** Strains expressing WT, K60,61R, SUMO-Tfg1, or mSUMO-Tfg1 forms of Tfg1-HA were used for Rpb1 IP followed by immunoblot analysis with HA and Rpb1 antibodies.

In yeast nuclear extracts, approximately 50% of RNAPII and 70% of TFIIF molecules are found in RNAPII-TFIIF complexes, which reflects a high level of physical and functional association between the two proteins [51]. We performed co-IP experiments to explore whether the RNAPII-TFIIF interaction is affected by Tfg1 sumoylation. IPs of WT and K60,61R forms of Tfg1-HA showed approximately equal levels of the large RNAPII subunit, Rpb1, and reciprocal IPs of Rpb1 showed equal levels of WT and K60,61R Tfg1-HA, indicating that Lys residues 60 and 61, or their SUMO modification, are not needed for the RNAPII-TFIIF interaction (Fig 5A and 5B). In IP analysis of Tfg1-HA derived from *ULP1* or *ulp1-mt* strains, however, elevated sumoylation of Tfg1 in the *ulp1-mt* strain correlated with significantly less IPed Rpb1 (Fig 5A). Similarly, in the reciprocal experiment, IP of Rpb1 resulted in dramatically less co-IP of Tfg1-HA in the *ulp1-mt* strain compared to in its *ULP1* wt parent strain (Fig 5B). This suggests that elevated levels of cellular sumoylation that result from the *ulp1-mt* mutation, or higher levels of Tfg1 sumoylation specifically, interfere with the interaction of TFIIF with RNAPII. To explore this further, we generated a strain that expresses, from the normal *TFG1* locus, Tfg1-HA with a non-cleavable, N-terminal fusion with the SUMO peptide, in order to somewhat mimic constitutively sumoylated Tfg1 (SUMO-Tfg1). The fusion-expressing strain shows only a modest growth defect, particularly at an elevated growth temperature, indicating that the N-terminal fusion does not substantially impair TFIIF function (S3E Fig). Importantly, however, HA and SUMO IP-immunoblot analysis shows that that sumoylation level of SUMO-Tfg1 is dramatically higher than that of WT Tfg1-HA (Fig 5C). This increased level of sumoylation is not entirely due to self polysumoylation of the fused SUMO moiety since highly elevated sumoylation was also detected in a version of this fusion in which all Lys residues are replaced with Arg in the SUMO moiety (mSUMO-Tfg1; Fig 5C). Intriguingly, Rpb1 IP analysis in the SUMO-Tfg1 and mSUMO-Tfg1 fusion strains shows significantly reduced interaction of the hyper-sumoylated forms of Tfg1 with Rpb1 compared to the interaction of Rpb1 with WT or K60,61R Tfg1 (Fig 5D). Although we recognize that the N-terminal SUMO peptide fusion does not necessarily recapitulate increased sumoylation of Tfg1 at its natural SUMO acceptor sites, this result, and the analysis performed in the *ulp1-mt* strain, support the idea that elevated Tfg1 sumoylation can reduce the interaction of TFIIF with RNAPII.

### Both reduced and constitutively elevated sumoylation at non-RPG promoters result in reduced RNAPII levels

**Fig 6:**
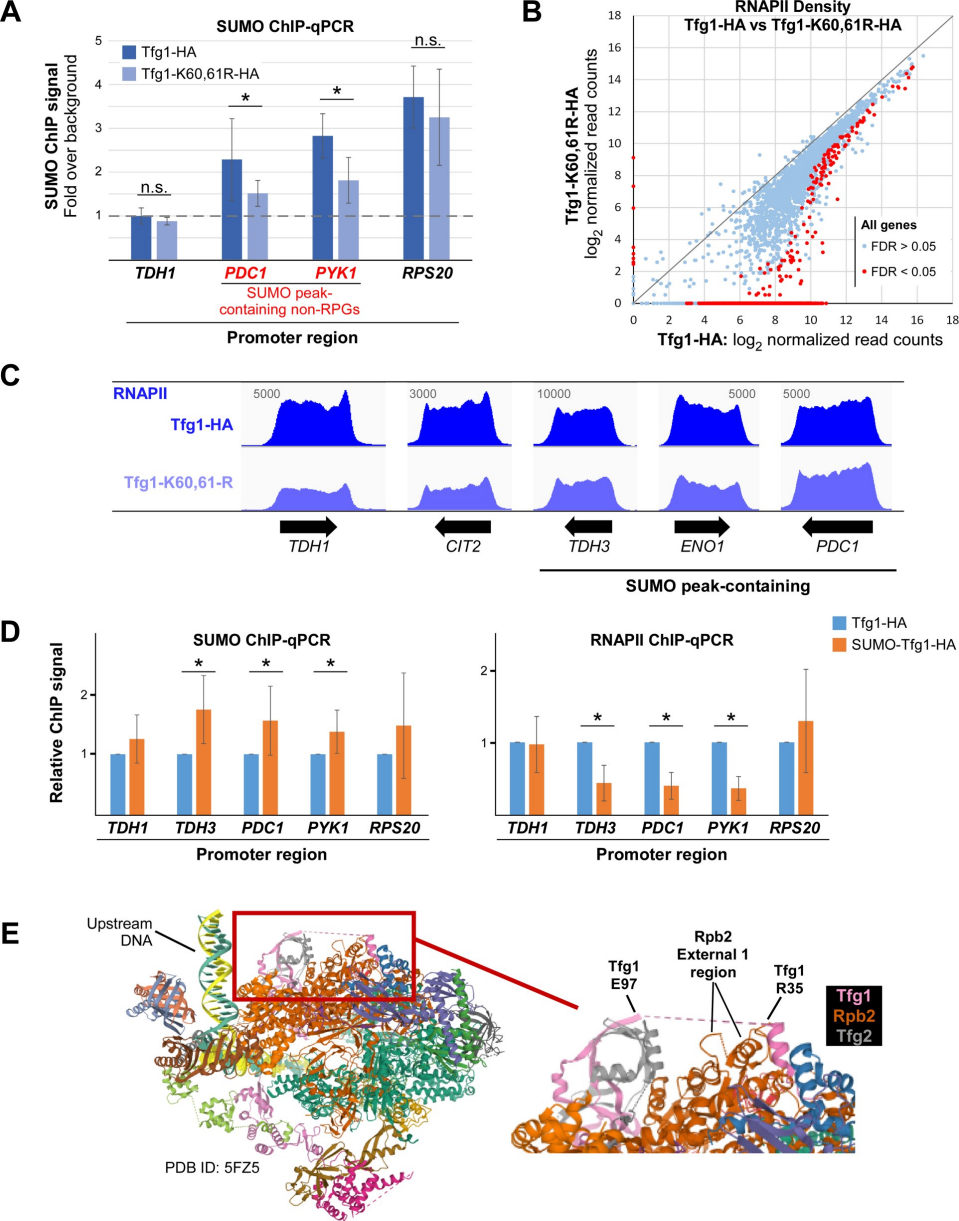
Both reducing and constitutively increasing promoter-associated SUMO levels can reduce RNAPII gene occupancy. **(A)** SUMO ChIP was performed in Tfg1-HA and Tfg1-K60,61R-HA strains followed by qPCR analysis of promoter regions of the indicated genes, including a non-RPG that lacks a significant SUMO peak, *TDH1*, two non-RPGs that contain a promoter-associated SUMO peak, *PDC1* and *PYK1* (also known as *CDC19*), and an RPG, *RPS20*. Quantification was performed by normalizing to the background ChIP signal for an untranscribed region of Chromosome V for which there is no detectable SUMO signal in the SUMO ChIP-seq analysis. Average and standard deviation of three replicates is shown, and Student’s *t*-tests showing significant difference (*p* value < 0.05) between paired samples are indicated with an asterisk. **(B)** RNAPII ChIP-seq was performed, with two independent replicates, in Tfg1-HA and Tfg1-K60,61R-HA strains, and RNAPII densities (log_2_ normalized read counts) were determined at each gene ORF and plotted. Differential binding analysis was performed, using the DiffBind tool, and genes showing significantly different levels of RNAPII (FDR < 0.05) in the two strains are indicated with red dots. See S12 Table for list of RNAPII densities by gene in Tfg1-HA and Tfg1-K60,61R-HA strains. **(C)** Sample RNAPII ChIP-seq alignments from Replicate 1 are shown for genes showing varying levels of differential RNAPII densities, including three non-RPGs that contain a prominent SUMO peak at their promoters. Values refer to maximum data range (read numbers) for the view shown, and unnormalized alignments were generated using IGV. **(D)** SUMO and RNAPII ChIP were performed in strains expressing Tfg1-HA or SUMO-Tfg1-HA, followed by qPCR analysis of promoter regions of the indicated genes. Average and standard deviation of three replicates is shown, and Student’s *t*-tests showing significant difference (*p* value < 0.05) between paired samples are indicated with an asterisk. **(E)** Position of Tfg1 Lys 60/61 within the structure of the yeast transcription initiation complex. Enlarged section at right shows the disordered 62-amino acid segment of Tfg1 (dashes between Arg 35 and Glu 97) within which lies Lys 60/61, and a proximal segment of Rpb2 including a loop and helix of the External 1 region. Tfg1 is represented by a pink ribbon, Rpb2 by dark orange, and Tfg2 by grey. Image generated with RCSB PDB (rcsb.org), with PDB ID: 5FZD [42].

To determine whether the SUMO peaks associated with promoters of non-RPGs derive from sumoylated Tfg1 specifically, we performed SUMO ChIP in Tfg1-HA and Tfg1-K60,61R-HA strains. The promoter regions of four genes were then analyzed by qPCR. *TDH1*, which is a non-RPG that does not have a SUMO peak according to our ChIP-seq analysis, showed no signal above background in the qPCR analysis, as expected (Fig 6A). For SUMO-peak containing non-RPGs *PDC1* and *PYK1* (*CDC19*), however, significant levels of SUMO were detected at their promoters, but this was substantially reduced in the Tfg1-K60,61R-HA strain. The sumoylation-impairing mutation of Tfg1 did not significantly reduce SUMO levels near the promoter of *RPS20*, which is an RPG that contains a SUMO peak associated with the position of Rap1 but not TBP (see Fig 1E). These data imply that sumoylation of promoter-associated TFIIF is a major contributor to the SUMO peaks associated specifically with non-RPGs. However, SUMO levels were not completely abolished in the Tfg1-K60,61R-HA strain, implying that other GTF components are also sumoylated at these promoters.

Next, we examined the effects of impaired Tfg1 sumoylation, and reduced promoter-associated sumoylation, on RNAPII levels genome-wide. RNAPII ChIP-seq was performed in Tfg1-HA and Tfg1-K60,61R strains, and RNAPII densities across all ORFs were determined. As shown in Figs 6B and S4B and S12 Table, strikingly, nearly half of all genes showed significantly reduced RNAPII occupancy in the Tfg1-K60,61R-HA strain, suggesting that Tfg1 sumoylation has a general positive effect on transcription. As we observed in the *ubc9-6* analysis, changes in RNAPII levels due to the Tfg1-K60,61R mutation vary widely by gene (examples in Fig 6C) and are not correlated with the presence or absence of detectable promoter-associated SUMO peaks (S12 Table and S4A Fig). This demonstrates that the effects of GTF sumoylation can be widespread and supports the possibility that promoter-associated sumoylation occurs at far more non-RPGs than we were able to detect. The reduction in RNAPII density was not sufficient to alter steady state mRNA levels for the vast majority of genes, however, as determined by RNA-seq analysis in the Tfg1-HA and Tfg1-K60,61R-HA strains (S4C Fig and S13 and S14 Tables). For example, in the Tfg1-K60,61R-HA strain, RNAPII levels were reduced on *LYS1* and *LYS2*, both involved in Lys biosynthesis, but steady-state mRNA levels for these genes were unaffected, and, correspondingly, the strain does not show Lys auxotrophy (S14 Table and S4D Fig). Nonetheless, together, these results demonstrate that Tfg1 sumoylation can be important for maintaining normal levels of chromatin-associated RNAPII at numerous genes but, as discussed below, simultaneous sumoylation of multiple GTF components, in addition to Tfg1, may be necessary in order to fully impact transcription.

Finally, to explore the effects of elevated levels of sumoylation at promoters of non-RPGs, we used the SUMO-Tfg1-HA strain, in which we expect SUMO to be tethered to active promoters through constitutive attachment to Tfg1. Compared to the Tfg1-HA strain, SUMO ChIP-qPCR analysis in the fusion strain showed significantly elevated levels of sumoylation associated with promoters of *TDH3*, *PDC1*, and *PYK1*, all of which normally have a detectable SUMO peak (S9 Table), whereas *TDH1* and the RPG *RSP20* did not show significantly elevated SUMO levels in this analysis (Fig 6D). Correspondingly, in RNAPII ChIP-qPCR analysis of these strains, genes that showed elevated promoter-associated sumoylation in the SUMO-Tfg1-HA strain also showed significantly reduced RNAPII levels, suggesting that constitutively elevated sumoylation at core promoters can impair transcription (Fig 6D). These results, together with the data described above, demonstrate that situations in which GTF sumoylation is blocked (through the Tfg1-K60/61-R mutation) and in which GTF sumoylation is constitutive (through SUMO fusion to Tfg1) can both reduce RNAPII occupancy levels on non-RPGs. As explained below, this implies that dynamic sumoylation is needed for normal transcription of many genes.

## Discussion

To date, nearly 600 proteins in budding yeast have been identified as targets of sumoylation, and our fractionation analysis demonstrates that most sumoylated proteins associate with chromatin ([8–10], and our data). Supporting previous work in yeast and human cells [38–40], our SUMO ChIP-seq analysis shows that the bulk of chromatin-bound sumoylated proteins are found at RPGs and tRNA genes, but we also detected 147 high-stringency SUMO peaks at promoters of non-RPGs. Chymkowitch and colleagues demonstrated that, in yeast, SUMO associated with RPGs derive from the sumoylated transcription factor Rap1, whereas SUMO at tRNA genes likely arises from sumoylated subunits of RNAPIII [23,40]. Our analysis now adds to this by providing strong evidence that SUMO peaks associated with promoters of non-RPGs derive from sumoylated GTFs, particularly TFIIF. As such, surprisingly, nearly 90% of all SUMO peaks associated with chromatin may be primarily attributed to Rap1, RNAPIII, and RNAPII GTF components. This leads to the question of why hundreds of other SUMO-modified chromatin-associated proteins were not detected by SUMO ChIP-seq.

A possible explanation is that the association of most sumoylated proteins with chromatin is highly transient and therefore difficult to capture by ChIP. That sumoylation can promote the dissociation of target proteins from chromatin has been observed with many SSTFs. For example, sumoylation of DNA-bound Gcn4, a yeast SSTF, and human c-Fos triggers their clearance from DNA to limit transcriptional activation of target genes [13,14,52]. Similarly, ChIP-based studies have shown that impairing the sumoylation of multiple other yeast and mammalian SSTFs leads to an increase in their occupancy levels at normal binding sites and/or binding to numerous non-specific additional sites across the genome [3,16–20,22]. Whereas SUMO is stably associated with DNA-bound Rap1, RNAPIII, or GTFs at many loci, these may be exceptions. Our current analysis supports the idea that, somewhat paradoxically, SUMO more generally targets chromatin-associated proteins in order to restrict or destabilize their association with chromatin, such that the occurrence of chromatin-bound SUMO-modified proteins is widespread, but highly transient [3,22].

Of over 5800 non-RPGs in yeast, most of which show at least some level of RNAPII occupancy during normal growth, why is SUMO detected at promoters of only 155? Our GO analysis indicates that about a quarter of these are involved in the biosynthesis of small molecules, suggesting that these metabolic pathways in particular are subject to regulation by sumoylation at the level of transcription. However, we also determined that SUMO peak-containing non-RPGs generally show high levels of RNAPII occupancy, which likely reflect high rates of transcription. This is consistent with studies in human cells, which found SUMO1 or SUMO2/3 enriched particularly at promoter regions of highly-expressed genes [38,39]. The detection of promoter associated sumoylation, therefore, might simply be a consequence of the relatively high levels of GTFs assembled on highly transcribed genes. This is supported by our finding that most SUMO peak-containing non-RPGs have high levels of TFIIF at their promoters, and it suggests that promoter-associated sumoylation is far more widespread, but difficult to detect. Indeed, in their SUMO ChIP-seq analysis, Chymkowitch and colleagues were able to detect stringent SUMO peaks at only 12 non-RPGs [40], whereas our more sensitive analysis revealed 147 non-RPG SUMO peaks, and future approaches are likely to reveal a more complete picture of the SUMO landscape across non-RPGs. A wider role for GTF sumoylation at promoters of non-RPGs is further supported by our finding that impairing global sumoylation, or blocking sumoylation of Tfg1 specifically, impacts transcription of far more genes than those with detectable SUMO peaks.

The difficulty in detecting SUMO at non-RPGs promoters may be related to the highly labile nature of the modification [45]. In support of this, we found that partially inactivating the SUMO protease Ulp1 resulted in increased sumoylation of chromatin-associated Tfg1, suggesting that the protein is normally subject to some level of desumoylation. Furthermore, the other major yeast SUMO protease, Ulp2, was recently shown to associate with active genes where it is thought to regulate a cascade of histone post-translational modifications and promote transcription elongation [53]. The presence of Ulp2 at promoters of active genes, however, suggests that it may also target sumoylated GTFs, resulting in low levels of sumoylation at most non-RGP promoters genome-wide. Further work will be needed to determine, perhaps by inactivating SUMO proteases, whether sumoylated GTFs can be detected as a more general feature of RNAPII transcription, and by what mechanism sumoylation is stabilized at the 147 non-RPG SUMO-peaks.

SUMO peaks associated with non-RPGs lie immediately upstream of their TSSs, precisely where GTFs assemble and distinct from the binding position of Mediator, SSTFs, and the SAGA complex, all of which generally interact with upstream activation sequences (UASs; [54]). Consistent with several proteomic analyses, we detected sumoylated proteins associated with four GTFs, TFIIA, TFIID, TFIIF, and TFIIE. Impairing sumoylation of one component, Tfg1, resulted in reduced RNAPII density across many genes, implying that there was an impact on transcription, but the effect was not sufficient for altering steady-state mRNA levels in most cases. Simultaneous SUMO modification of multiple components of the promoter-assembled PIC might be necessary to synergistically impart a more significant effect on transcription. Referred to as protein-group sumoylation, such a phenomenon has been observed with protein complexes involved in DNA repair [55]. How protein-group sumoylation regulates promote-bound GTFs remains to be determined but will require identifying additional targets and studying the effects of impairing their sumoylation individually and as a group.

For Tfg1, we determined that Lys 60/61 is the major site of Tfg1 sumoylation. Intriguingly, both acetylation and ubiquitination have been detected at Lys 61 in some conditions, suggesting a possible interplay among post-translational modifications at this site, and indicating that this region of the protein is subject to much regulation [56,57]. When examined as part of the structure of GTFs assembled on promoter DNA with RNAPII, Lys 60/61 lies in an exposed area of the complex, within a 62-amino acid disordered segment of Tfg1 (Fig 6E; [42]). Proximal to the presumed position of Lys 60/61 of Tfg1 is a loop and helix of the External 1 region of the second-largest subunit of RNAPII, Rpb2 [58]. Based on this proximity, and supported by our data that show that elevated Tfg1 sumoylation correlates with reduced TFIIF-RNAPII association, we speculate that SUMO modification of Lys 60/61 modulates the interaction of TFIIF with RNAPII.

What effect does GTF sumoylation have on transcription of non-RPGs? Partially inactivating the sole SUMO conjugating enzyme, Ubc9, which virtually eliminated promoter-associated sumoylation, reduced RNAPII occupancy levels at highly transcribed SUMO peak-containing non-RPGs in both this genome-wide study and in a previous analysis of three constitutively expressed genes, *PYK1*, *ADH1*, and *PMA1* [37]. This, and the finding that the most highly transcribed genes are more likely to have a promoter-associated SUMO peak than other genes, points to a positive role for sumoylation in transcription. Nonetheless, many moderately or lowly transcribed SUMO peak-containing non-RPGs showed unaltered or elevated RNAPII levels in the *ubc9-6* mutant strain. Similarly in human cells, depletion of the SUMO1 isoform led to downregulation specifically of highly-expressed genes that normally contain SUMO1-labeled proteins at their promoters, whereas less expressed genes were generally upregulated by the depletion [38]. The effects of inactivating Ubc9, though, are likely complex, since it causes a global reduction of SUMO conjugation, making it difficult to attribute changes in RNAPII levels to decreased sumoylation of promoter-bound GTFs, specifically.

Our analysis of Tfg1, however, also supports a positive role for GTF sumoylation in regulating transcription. For one, SUMO site mutations on Tfg1, which partly reduce promoter-associated sumoylation, caused a general reduction in RNAPII levels across many genes. On the other hand, fusing SUMO to Tfg1, which elevated SUMO levels at promoters of some genes, also resulted in reduced RNAPII levels at those genes. Although these results may seem contradictory, we believe they indicate that dynamic GTF sumoylation, more so than simply the presence or absence of SUMO-modified GTF components, is needed for normal transcription of many genes, and we propose the following model based on our results. Firstly, preferentially nonsumoylated TFIIF associates with RNAPII prior to recruitment of the TFIIF-RNAPII sub-complex to PICs forming at promoters of non-RPGs. This is consistent with our observation that impairing Tfg1 sumoylation does not affect the association of TFIIF with RNAPII. Once the complete PIC is formed, sumoylation of Tfg1 and other GTF components at some genes, including many highly transcribed genes, then triggers rearrangements of the PIC that lead to increased transcription by as-of-yet unknown mechanisms. Sumoylation of Tfg1 specifically might facilitate its dissociation from PICs, including from RNAPII, prior to elongation, thereby resetting promoters for further rounds of TFIIF-RNAPII recruitment (i.e. reinitiation; [59]) and increased transcription. This hypothesis aligns with our previously proposed general role for sumoylation in enhancing the dissociation of SSTFs from chromatin and with our data presented here that demonstrate that elevated Tfg1 sumoylation significantly reduces its interaction with RNAPII [22]. Additionally, by reducing the TFIIF-RNAPII interaction, fusion of SUMO to Tfg1 would be expected to inhibit transcription, as we have observed, by limiting the number of TFIIF-RNAPII complexes available for PIC formation. Further work is needed to explore this model, but taken together, our data support the idea that dynamic sumoylation of GTFs functions to positively regulate transcription at many non-RPGs. Together with work published in 2005 on sumoylation of human TFIID [29], our study is among the first to examine the effects of GTF sumoylation, laying the groundwork for future studies which are needed to fully understand how SUMO modification regulates the complex dynamics of the promoter-associated transcription machinery across eukaryotes.

## Methods

### Yeast strains and growth medium

Yeast strains used in this study are listed in S1 Table. Yeast cultures were grown in synthetic complete (SC) medium at 30°C to mid-log phase, unless otherwise noted. Yeast growth assays on solid medium (spot assays) were performed by dropping approximately 10,000 cells onto the first spot with serial five-fold dilutions in adjacent positions, as previously described [14]. Epitope tagging and mutagenesis were performed at natural target gene loci by homologous recombination-based transformation with PCR-generated linear DNA fragments that contain selective markers [60].

The *ubc9-6* allele is a tandem repeat of the *ubc9-1* allele integrated at the *UBC9* locus. This increases the abundance the Ubc9-1 protein in cells and alleviates the slow growth phenotype of the original *ubc9-1* mutant at permissive temperature (as well as the propensity of the strain to accumulate an extra copy of chromosome III where the original *ubc9-1* allele was integrated; [61]). To create the *ubc9-6* mutant yeast strain (D370), a fragment encoding *ubc9-1*:*Tadh1*:*TRP1*:*ubc9-1* was subcloned in a pFA6a-derived plasmid. The fragment was excised from this vector using PacI-EcoRI, transformed into a W303 background strain and the resulting transformants selected for integration of *ubc9-6* at the endogenous locus. The exact sequence of the locus can be obtained on request.

The SUMO-Tfg1 fusion strain was made by first generating a DNA fragment, using fusion PCR, consisting of the coding sequence the for yeast SUMO (lacking the sequence encoding the C-terminal GGATY motif), the *TFG1* ORF, the 6xHA epitope tag sequence with a stop codon, and the *K*. *lactis TRP1* marker cassette, flanked with sequences that target the DNA fragment to the *TFG1* locus by homologous recombination. The fragment was then transformed into a *TFG1/tfg1Δ*::*URA3* heterozygous diploid strain of the W303 background, transformants resistant to 5-fluoroorotic acid (5-FOA) were selected and sporulated, and TRP+ meiotic progeny were isolated and confirmed to contain the fusion construct at the *TFG1* locus by PCR and sequencing. The mSUMO-Tfg1 strain was generated by the same method, except that a yeast SUMO sequence in which all Lys codons are replaced by Arg codons (KallR) was used. Late in the study, it was noticed that the Tfg1-K60,61R strain also carries another, inadvertent mutation that results in the missense substitution N462Q. However, this is a conserved mutation on a residue that is not near a putative sumoylation site, and is therefore not likely to affect Tfg1 sumoylation or function.

### Immunoprecipitation and immunoblots

For IP analysis, cultures were grown to mid-log phase, then lysates were prepared in non-denaturing conditions as described previously [20]. Briefly, cell pellets were washed then resuspended with ice-cold IP buffer (50 mM Tris-HCl, pH 8; 150 mM NaCl; 0.1% Nonidet P-40 (NP40); 1 mM phenylmethylsulphonyl fluoride (PMSF); and 2.5 mg/mL *N*-ethylmaleimide (NEM)); yeast protease inhibitor cocktail (BioShop)). Samples were vortexed with acid-washed glass beads, then lysates were clarified by two rounds of centrifugation. Clarified lysates were incubated overnight with Protein G agarose beads and 1 μg of the appropriate antibody: HA or Rpb1, as listed below. Beads were washed with IP buffer three times, then once in IP buffer lacking NP40. Finally, IPed proteins were eluted by boiling the beads for 4 min in 2X sample buffer (140 mM Tris-HCl, pH 6.8; 4% SDS; 20% glycerol; 0.02% bromophenol blue; supplemented with 10% 2-mercaptoethanol prior to use). Soluble material was then analyzed by SDS polyacrylamide gel electrophoresis using standard protocols, and most immunoblots were visualized using a MicroChemi chemiluminescence imager (DNR). Antibodies used for immunoblots are: 1:500 yeast SUMO/Smt3 (y-84; Santa Cruz, sc-28649); 1:3000 GAPDH (Sigma, G9545); histone H3 (1:3000 for yeast immunoblot, 1:20,000 for HeLa immunoblot; Abcam, ab1791); 1:500 SUMO1 (Developmental Studies Hybridoma Bank, 21C7); 1:50 SUMO2/3 (Developmental Studies Hybridoma Bank, 8A2); 1:0000 α-tubulin (Cell Signaling Technology, 2144); 1:1000 HA (Novus, NB600-363); 1:1000 Rpb1 (8WG16; Abcam, ab817); and 1:1000 Rpb3 (Abcam; ab202893).

### Chromatin fractionation

For yeast chromatin fractionation, whole cell extracts, soluble extracts, and low-salt chromatin fractions were isolated using a previously described protocol [62]. HeLa cell fractionation was performed as previously described [63]. Fractions were then analyzed by SUMO immunoblots with control immunoblots for chromatin-associated (histone H3) or cytoplasmic proteins (GAPDH or α-tubulin).

### ChIP and ChIP-seq

Small-scale ChIP, with 50-mL cultures, and subsequent qPCR analysis, were performed as previously described [20], using 10 μL of the yeast SUMO/Smt3 antibody described above. qPCR primer sequences are listed in S8 Table. For ChIP-seq, 200-mL cultures were grown in SC medium and the ChIP protocol was scaled up accordingly, with the following antibody volumes used per IP in each experiment: for SUMO ChIP-seq, 8 μg of yeast SUMO antibody (y-84; Santa Cruz, sc-28649); for RNAPII ChIP-seq, 15 μg of the Rpb1 antibody (8WG16; Abcam, ab817). Details of library preparation, sequencing and bioinformatics analysis for the three ChIP-seq experiments are listed in S4, S5 and S6 Tables. SUMO peak sets and RNAPII ORF densities obtained from analysis of the ChIP-seq experiments are listed in S9, S11 and S12 Tables. Statistics for the SUMO ChIP-seq and the RNAPII ChIP-seq in the Tfg1-HA and Tfg1-K60,61R-HA strains are presented in S2 and S3 Tables, respectively. Further comprehensive analysis of the RNAPII ChIP-seq performed in WT and *ubc9-6* strains will be presented elsewhere (Moallem, Bergey, and Rosonina, manuscript in preparation). When comparing ChIP-seq data between samples, statistical significance was determined by a false discovery rate (FDR) of less than 0.5.

### Preparation of RNA and RNA-seq

Total RNA was prepared as follows. 10-mL cultures growing at mid-log phase in SC medium were pelleted, washed with 1 mL of ice-cold AE buffer (50 mM sodium acetate, pH 5.2; 10 mM EDTA, pH 8), pelleted again, then resuspended in 400 μL of ice-cold AE and 40 μL of 10% SDS. An equal volume of phenol, pH 4.5 was added, and the samples were mixed thoroughly, placed in a dry ice/ethanol slurry for 5 min, transferred to a 65°C water bath for 5 min, then vortexed for 30 s. Another freeze-heat-vortex-freeze cycle was performed, then samples were centrifuged at top microfuge speed for 7 min at room temperature. To the aqueous layer, 600 μL of a 1:1 phenol (pH 4.5)-chloroform mixture was added, samples were vortexed, then centrifuged for 5 min. RNA in the aqueous layer was then precipitated by adding 50 μL of 3 M sodium acetate (pH 5.2) and 1 mL of ice-chilled absolute ethanol, incubating on dry ice for 10 min, then centrifuging for 15 min at 4°C. Pellets were washed with ice-cold 70% ethanol, then resuspended in 100 μL of nuclease-free water. For RNA-seq, RNA samples were then further purified using the RNeasy kit (Qiagen), and eluted with 100 μL of nuclease free water. Polyadenylated (polyA) RNAs were enriched prior to sequencing. Details of polyA enrichment, library preparation, sequencing, and bioinformatics analysis are listed in S7 Table. For qPCR analysis, 1 μg of DNase-treated RNA was reverse transcribed using the iScript cDNA synthesis kit (BioRad), and primer sequences are indicated in S8 Table.

## Supporting information

S1 Fig Supporting data for SUMO and RNAPII ChIP-seq analyses. **(A)** Venn diagram showing comparison of unedited peak sets from the two independent SUMO ChIP-seq replicates. **(B)** Venn diagram showing comparison of the high stringency SUMO ChIP-seq peak set from this study and the high stringency Flag-SUMO ChIP-seq peak set from a previous study [40]. The current study identified 236 high stringency SUMO peaks that were not identified in the previous study, 136 of which are associated with non-RPGs. **(C)** Levels of SUMO peaks do not correlate with transcription level, as approximated by RNAPII densities. Scatter plot of SUMO peak level versus RNAPII density for 111 non-RPGs with a unique SUMO peak. Pearson coefficient (*r*) implies no significant correlation. **(D)** The *ubc9-6* strain has dramatically reduced SUMO conjugation levels. SUMO and GAPDH immunoblots of lysates prepared from *ubc9-6* and its parental strain (WT; W303a). All SUMO-peaks at non-RPGs were dramatically reduced in *ubc9-6* (see Fig 2C and 2D). **(E)** Spot assays examining growth of the *ubc9-6* strain on SC medium at the permissive temperature (30°), in which experiments in this study were performed, or at the non-permissive temperature of 37°C. **(F)** Validation of RNAPII ChIP-seq. Occupancy values obtained from DiffBind analysis of the two ChIP-seq replicates for WT and *ubc9-6* strains across ORFs of selected genes is shown at top. An independent RNAPII ChIP was performed in the same strains and qPCR analysis of the promoter region of the same genes is shown at bottom. Genes analyzed are a selection of SUMO peak-containing genes that include those with high RNAPII density (*TDH3*, *PGK1*, *ENO1*) and modest RNAPII density (*ALD6*, *YHB1*, *ACT1*), as determined by our ChIP-seq analyses. Asterisks (*) indicate genes with significantly different RNAPII occupancy levels in the two strains (FDR < 0.05) according to the DiffBind analysis (see S5 Table for details).
(PDF)

S2 Fig Motif analysis of SUMO peak sequences. **(A)** Motif analysis of SUMO peak-containing sequences. MEME analysis, for identifying novel, ungapped, recurring patterns [47], was used with 100-nt sequences encompassing 118 RPG-associated SUMO peaks or 133 SUMO peaks associated with non-RPGs that do not contain a known Rap1 binding site. The top motif identified for the RPG set is shown, which matches the known Rap1 binding site consensus (also shown). Only one motif was identified for the non-RPG set, as shown. **(B)** Motif analysis using DREME, from the MEME suite of tools, was applied to identify short (8-nt or less) motifs that are relatively enriched, using 300-nt sequences encompassing the RPG or non-RPG SUMO peak sets described in *A*. The top result is shown for the RPG set, which matches the Rap1 binding motif (see *A*). Both significant results produced for the non-RPG set are shown, with the top result indicating that 94 of the 133 sequences contain a TATA box element (consensus TATAWAWR). **(C)** Multiple subunits of GTFs and RNAPII are putative SUMO targets (shaded in blue), based on published proteomics analyses. Note that TAF14, also known as Tfg3, is also considered a subunit of yeast TFIIF. References are as follows: a, [30]; b, [31]; c, [32]; d, [33]; e, [8]; f, [10]. Note that only Tfg1 has been identified all six studies. **(D)** Genomic sites with high levels of TFIIF generally show high levels of SUMO. TFIIF ChIP-seq peaks across the genome were identified (for its Tfg2 subunit) and plotted by increasing raw read count values (based on data obtained from GEO database accession number GSM4319120). Raw read counts from our SUMO ChIP-seq analysis at each of the corresponding TFIIF peaks were determined and plotted above.
(PDF)

S3 Fig Analysis of Tfg1 sumoylation. **(A)** Arg substitution of Lys 60,61, but not Lys 91, 658, or 733, significantly impairs sumoylation of Tfg1. Tfg1-HA-expressing strains with the indicated Lys-to-Arg substitutions, or unmodified (WT), were used in HA IP experiments followed by SUMO and HA immunoblots to examine the effects of the mutations on Tfg1 sumoylation levels. **(B)** Impairing Tfg1 sumoylation is not sufficient to affect growth. Strains expressing Tfg1-HA in its wild-type form (WT), or with K60,61R or K91R mutations, and the parental, unmodified lab strain W303a, were used in a spot assay, comparing growth on rich medium (YPD), synthetic medium (SC), or synthetic medium lacking tryptophan (SC-TRP). Strains expressing HA-tagged Tfg1 are marked with the *Kluyveromyces lactis TRP1* gene. **(C)** Liquid cultures of WT or K60,61R version of Tfg1-HA strains were prepared at an absorbance (595 nm) of ~0.2, then culture absorbance measurements were taken over a 20 h period using an accuSkan absorbance microplate reader (Fisherbrand). Culture density values are arbitrary. Average of three experiments is shown; error bars representing standard deviation are small and obscured by the thickness of the curves. **(D)** Spot assays were performed to compare growth of strains expressing WT or K60,61R forms of Tfg1-HA on SC medium in normal conditions or in the presence of the indicated stressors. Normal yeast growth temperature is 30°C. **(E)** Spot assays were performed on YPD or SC medium at 30°C or 37°C using unmodified lab strains W303a and W303α, a strain expressing WT Tfg1-HA, and a strain expressing Tfg1-HA with an N-terminal fusion to the yeast SUMO peptide (Smt3 residues 1–96, lacking the protease-targeting C-terminal GG motif). **(F)** Liquid growth curves were generated for *ULP1* and *ulp1-mt* strains, as in S3C Fig.
(PDF)

S4 Fig Effects of altered Tfg1 sumoylation on RNAPII density and steady-state RNA levels. **(A)** Scatterplot showing changes in RNAPII densities for non-RPGs associated with unique SUMO peaks in Tfg1-HA versus Tfg1-K60,61R-HA strains, sorted horizontally by SUMO peak levels. Genes showing a significant difference, as determined by differential binding analysis with DiffBind, are represented by red dots. **(B)** Validation of RNAPII ChIP-seq. Occupancy values obtained from DiffBind analysis of the two ChIP-seq replicates for Tfg1-HA and Tfg1-K60,61R-HA strains across ORFs of selected genes is shown at top. An independent RNAPII ChIP was performed in the same strains and qPCR analysis of the promoter region of the same genes is shown at bottom. Genes analyzed are a selection of SUMO peak-containing genes that include those with high RNAPII density (*TDH3*, *PGK1*, *ENO1*) and modest RNAPII density (*ALD6*, *YHB1*, *ACT1*), as determined by our ChIP-seq analyses. Asterisks (*) indicate genes with significantly different RNAPII occupancy levels in the two strains (FDR < 0.05) according to the DiffBind analysis (see S6 Table for details). **(C)** RNA-seq analysis was performed in Tfg1-HA and Tfg1-K60,6R-HA strains, and differential expression analysis (using edgeR) is plotted, as log_2_ of the ratio of expression in the two strains, sorted horizontally by average log_2_ expression (in counts per million, CPM). Genes showing significantly higher RNA abundance in Tfg1-K60,61R-HA are represented with red dots, and those showing significantly lower RNA abundance in that strain are shown in blue. See S13 Table for results of RNA-seq analysis. At bottom, an independent set of RNAs was obtained from the same strains and RT-qPCR was performed on a selection of genes, which also shows no significant difference in steady-state RNA levels. **(D)** Reduced global sumoylation or impaired Tfg1 sumoylation does not result in Lys auxotrophy. Spot assay comparing growth of indicated strains on SC medium and SC medium lacking lysine.
(PDF)

S1 Table Yeast strains used in this study. (PDF)

S2 Table Peak set statistics for SUMO ChIP-seq. (PDF)

S3 Table Statistics for RNAPII ChIP-seq and RNA-seq in Tfg1-HA vs. Tfg1-K60,61R strains. (PDF)

S4 Table Analysis details for SUMO ChIP-seq. (PDF)

S5 Table Analysis details for RNAPII ChIP-seq in WT vs. *ubc9-6* strains. (PDF)

S6 Table Analysis details for RNAPII ChIP-seq in Tfg1-HA vs. Tfg1-K60,61R strains. (PDF)

S7 Table Analysis details for RNA-seq in Tfg1-HA vs. Tfg1-K60,61R-HA strains. (PDF)

S8 Table Sequences of oligonucleotides used in this study. (PDF)

S9 Table SUMO peak list. (XLSX)

S10 Table GO term analysis of SUMO peak list. (XLSX)

S11 Table RNAPII ORF density list for WT vs. *ubc9-6* strains. (XLSX)

S12 Table RNAPII ORF density list for Tfg1-HA vs. Tfg1-K60,61R-HA strains. (XLSX)

S13 Table RNA levels list for Tfg1-HA vs. Tfg1-K60,61R strains. (XLSX)

S14 Table Genes affected by the tfg1-K60,61R mutation. (XLSX)

S15 Table Numerical data for graphs. (XLSX)

## References

[1] Flotho A, Melchior F. Sumoylation: A Regulatory Protein Modification in Health and Disease. Annu Rev Biochem. 2013;82: 357–385. doi: 10.1146/annurev-biochem-061909-093311 23746258

[2] Boulanger M, Chakraborty M, Tempé D, Piechaczyk M, Bossis G. SUMO and Transcriptional Regulation: The Lessons of Large-Scale Proteomic, Modifomic and Genomic Studies. Molecules. 2021;26: 828. doi: 10.3390/molecules26040828 33562565 PMC7915335

[3] Rosonina E, Akhter A, Dou Y, Babu J, Sri Theivakadadcham VS. Regulation of transcription factors by sumoylation. Transcription. 2017;8: 220–231. doi: 10.1080/21541264.2017.1311829 28379052 PMC5574528

[4] Chymkowitch P, Nguéa P A, Enserink JM. SUMO-regulated transcription: Challenging the dogma. BioEssays. 2015;37: 1095–1105. doi: 10.1002/bies.201500065 26354225

[5] Zhao X. SUMO-Mediated Regulation of Nuclear Functions and Signaling Processes. Mol Cell. 2018;71: 409–418. doi: 10.1016/j.molcel.2018.07.027 30075142 PMC6095470

[6] Hendriks IA, Lyon D, Young C, Jensen LJ, Vertegaal ACO, Nielsen ML. Site-specific mapping of the human SUMO proteome reveals co-modification with phosphorylation. Nat Struct Mol Biol. 2017 [cited 3 Feb 2017]. doi: 10.1038/nsmb.3366 28112733

[7] Nayak A, Müller S. SUMO-specific proteases/isopeptidases: SENPs and beyond. Genome Biol. 2014;15: 422. doi: 10.1186/s13059-014-0422-2 25315341 PMC4281951

[8] Albuquerque CP, Yeung E, Ma S, Fu T, Corbett KD, Zhou H. A Chemical and Enzymatic Approach to Study Site-Specific Sumoylation. Edelmann MJ, editor. PLoS One. 2015;10: e0143810. doi: 10.1371/journal.pone.0143810 26633173 PMC4669148

[9] Makhnevych T, Sydorskyy Y, Xin X, Srikumar T, Vizeacoumar FJ, Jeram SM, et al. Global map of SUMO function revealed by protein-protein interaction and genetic networks. Mol Cell. 2009;33: 124–135. doi: 10.1016/j.molcel.2008.12.025 19150434

[10] Esteras M, Liu I-C, Snijders AP, Jarmuz A, Aragon L. Identification of SUMO conjugation sites in the budding yeast proteome. Microb Cell. 2017;4: 331–341. doi: 10.15698/mic2017.10.593 29082231 PMC5657824

[11] Escobar-Ramirez A, Vercoutter-Edouart A-S, Mortuaire M, Huvent I, Hardivillé S, Hoedt E, et al. Modification by SUMOylation Controls Both the Transcriptional Activity and the Stability of Delta-Lactoferrin. PLoS One. 2015;10: e0129965. doi: 10.1371/journal.pone.0129965 26090800 PMC4474976

[12] Yang S-HH, Sharrocks AD. SUMO promotes HDAC-mediated transcriptional repression. Mol Cell. 2004;13: 611–617. doi: 10.1016/s1097-2765(04)00060-7 14992729

[13] Rosonina E, Duncan SM, Manley JL. Sumoylation of transcription factor Gcn4 facilitates its Srb10-mediated clearance from promoters in yeast. Genes Dev. 2012;26: 350–355. doi: 10.1101/gad.184689.111 22345516 PMC3289883

[14] Akhter A, Rosonina E. Chromatin association of gcn4 is limited by post-translational modifications triggered by its DNA-binding in Saccharomyces cerevisiae. Genetics. 2016;204: 1433–1445. doi: 10.1534/genetics.116.194134 27770033 PMC5161277

[15] Sutinen P, Rahkama V, Rytinki M, Palvimo JJ. Nuclear Mobility and Activity of FOXA1 with Androgen Receptor Are Regulated by SUMOylation. Mol Endocrinol. 2014;28: 1719–1728. doi: 10.1210/me.2014-1035 25127374 PMC5414787

[16] Bertolotto C, Lesueur F, Giuliano S, Strub T, de Lichy M, Bille K, et al. A SUMOylation-defective MITF germline mutation predisposes to melanoma and renal carcinoma. Nature. 2011;480: 94–8. doi: 10.1038/nature10539 22012259

[17] Paakinaho V, Kaikkonen S, Makkonen H, Benes V, Palvimo JJ. SUMOylation regulates the chromatin occupancy and anti-proliferative gene programs of glucocorticoid receptor. Nucleic Acids Res. 2014;42: 1575–1592. doi: 10.1093/nar/gkt1033 24194604 PMC3919585

[18] Paakinaho V, Lempiäinen JK, Sigismondo G, Niskanen EA, Malinen M, Jääskeläinen T, et al. SUMOylation regulates the protein network and chromatin accessibility at glucocorticoid receptor-binding sites. Nucleic Acids Res. 2021 [cited 8 Feb 2021]. doi: 10.1093/nar/gkab032 33524141 PMC7913686

[19] Sutinen P, Malinen M, Heikkinen S, Palvimo JJ. SUMOylation modulates the transcriptional activity of androgen receptor in a target gene and pathway selective manner. Nucleic Acids Res. 2014;42: 8310–8319. doi: 10.1093/nar/gku543 24981513 PMC4117771

[20] Sri Theivakadadcham VS, Bergey BG, Rosonina E. Sumoylation of DNA-bound transcription factor Sko1 prevents its association with nontarget promoters. PLoS Genet. 2019;15: e1007991. doi: 10.1371/journal.pgen.1007991 30763307 PMC6392331

[21] Zhang FP, Malinen M, Mehmood A, Lehtiniemi T, Jääskeläinen T, Niskanen EA, et al. Lack of androgen receptor SUMOylation results in male infertility due to epididymal dysfunction. Nat Commun. 2019;10: 1–12. doi: 10.1038/s41467-018-07882-8 30770815 PMC6377611

[22] Rosonina E. A conserved role for transcription factor sumoylation in binding-site selection. Curr Genet. 2019;65: 1–6. doi: 10.1007/s00294-018-0852-6 31093693

[23] Chymkowitch P, Nguéa P A, Aanes H, Robertson J, Klungland A, Enserink JM. TORC1-dependent sumoylation of Rpc82 promotes RNA polymerase III assembly and activity. Proc Natl Acad Sci U S A. 2017;114: 1039–1044. doi: 10.1073/pnas.1615093114 28096404 PMC5293095

[24] Nguéa P A, Robertson J, Herrera MC, Chymkowitch P, Enserink JM. De-sumoylation of RNA polymerase III lies at the core of the Sumo stress response in yeast. J Biol Chem. 2019; doi: 10.1074/jbc.RA119.009721 31676685 PMC6901327

[25] Wang Z, Wu C, Aslanian A, Yates JR, Hunter T. Defective RNA polymerase III is negatively regulated by the SUMO-ubiquitin-Cdc48 pathway. Elife. 2018;7. doi: 10.7554/eLife.35447 30192228 PMC6128692

[26] Chen X, Ding B, LeJeune D, Ruggiero C, Li S. Rpb1 sumoylation in response to UV radiation or transcriptional impairment in yeast. PLoS One. 2009;4: e5267. doi: 10.1371/journal.pone.0005267 19384408 PMC2668072

[27] Heckmann I, Kern MJ, Pfander B, Jentsch S. A SUMO-dependent pathway controls elongating RNA Polymerase II upon UV-induced damage. Sci Rep. 2019;9: 17914. doi: 10.1038/s41598-019-54027-y 31784551 PMC6884465

[28] Wykoff DD, O’Shea EK. Identification of sumoylated proteins by systematic immunoprecipitation of the budding yeast proteome. Mol Cell Proteomics. 2005;4: 73–83. doi: 10.1074/mcp.M400166-MCP200 15596868

[29] Boyer-Guittaut M, Birsoy K, Potel C, Elliott G, Jaffray E, Desterro JM, et al. SUMO-1 modification of human transcription factor (TF) IID complex subunits: inhibition of TFIID promoter-binding activity through SUMO-1 modification of hsTAF5. J Biol Chem. 2005;280: 9937–9945. M414149200 [pii] doi: 10.1074/jbc.M414149200 15637059

[30] Denison C, Rudner AD, Gerber SA, Bakalarski CE, Moazed D, Gygi SP. A proteomic strategy for gaining insights into protein sumoylation in yeast. Mol Cell Proteomics. 2005;4: 246–254. doi: 10.1074/mcp.M400154-MCP200 15542864

[31] Wohlschlegel JA, Johnson ES, Reed SI, Yates JR, Yates 3rd JR. Global analysis of protein sumoylation in Saccharomyces cerevisiae. J Biol Chem. 2004;279: 45662–45668. doi: 10.1074/jbc.M409203200 15326169

[32] Panse VG, Hardeland U, Werner T, Kuster B, Hurt E. A proteome-wide approach identifies sumoylated substrate proteins in yeast. J Biol Chem. 2004;279: 41346–41351. doi: 10.1074/jbc.M407950200 15292183

[33] Hannich JT, Lewis A, Kroetz MB, Li SJ, Heide H, Emili A, et al. Defining the SUMO-modified proteome by multiple approaches in Saccharomyces cerevisiae. J Biol Chem. 2005;280: 4102–4110. doi: 10.1074/jbc.M413209200 15590687

[34] Garcia-Dominguez M, Reyes JC. SUMO association with repressor complexes, emerging routes for transcriptional control. Biochim Biophys Acta. 2009;1789: 451–459. S1874-9399(09)00078-9 [pii] doi: 10.1016/j.bbagrm.2009.07.001 19616654

[35] Ouyang J, Gill G. SUMO engages multiple corepressors to regulate chromatin structure and transcription. Epigenetics. 2009;4: 440–444. 9807 [pii] doi: 10.4161/epi.4.7.9807 19829068

[36] Gill G. Something about SUMO inhibits transcription. Curr Opin Genet Dev. 2005;15: 536–541. doi: 10.1016/j.gde.2005.07.004 16095902

[37] Rosonina E, Duncan SM, Manley JL. SUMO functions in constitutive transcription and during activation of inducible genes in yeast. Genes Dev. 2010;24: 1242–1252. doi: 10.1101/gad.1917910 20504900 PMC2885660

[38] Liu HW, Zhang J, Heine GF, Arora M, Gulcin Ozer H, Onti-Srinivasan R, et al. Chromatin modification by SUMO-1 stimulates the promoters of translation machinery genes. Nucleic Acids Res. 2012;40: 10172–10186. doi: 10.1093/nar/gks819 22941651 PMC3488252

[39] Neyret-Kahn H, Benhamed M, Ye T, Le Gras S, Cossec JC, Lapaquette P, et al. Sumoylation at chromatin governs coordinated repression of a transcriptional program essential for cell growth and proliferation. Genome Res. 2013;23: 1563–1579. doi: 10.1101/gr.154872.113 23893515 PMC3787255

[40] Chymkowitch P, Nguéa AP, Aanes H, Koehler CJ, Thiede B, Lorenz S, et al. Sumoylation of Rap1 mediates the recruitment of TFIID to promote transcription of ribosomal protein genes. Genome Res. 2015;25: 897–906. doi: 10.1101/gr.185793.114 25800674 PMC4448685

[41] Rhee HS, Pugh BF. Genome-wide structure and organization of eukaryotic pre-initiation complexes. Nature. 2012;483: 295–301. doi: 10.1038/nature10799 22258509 PMC3306527

[42] Plaschka C, Hantsche M, Dienemann C, Burzinski C, Plitzko J, Cramer P. Transcription initiation complex structures elucidate DNA opening. Nature. 2016;533: 353–358. doi: 10.1038/nature17990 27193681

[43] Cubenas-Potts C, Matunis MJ, Cubeñas-Potts C, Matunis MJ. SUMO: a multifaceted modifier of chromatin structure and function. Dev Cell. 2013;24: 1–12. doi: 10.1016/j.devcel.2012.11.020 23328396 PMC3555686

[44] Hendriks IA, Vertegaal ACO. A comprehensive compilation of SUMO proteomics. Nat Rev Mol Cell Biol. 2016;17: 581–595. doi: 10.1038/nrm.2016.81 27435506

[45] Moallem M, Akhter A, Babu J, Burke GL, Khan F, Bergey BG, et al. Normal levels of cellular sumoylation are largely dispensable for growth but facilitate heat tolerance in yeast. bioRxiv. bioRxiv; 2019. p. 761759. doi: 10.1101/761759

[46] Tan K, Wong KH. RNA polymerase II ChIP-seq-a powerful and highly affordable method for studying fungal genomics and physiology. Biophys Rev. 2019;11: 79–82. doi: 10.1007/s12551-018-00497-9 30627870 PMC6381363

[47] Bailey TL, Johnson J, Grant CE, Noble WS. The MEME Suite. Nucleic Acids Res. 2015;43: W39–W49. doi: 10.1093/nar/gkv416 25953851 PMC4489269

[48] Eyboulet F, Wydau-Dematteis S, Eychenne T, Alibert O, Neil H, Boschiero C, et al. Mediator independently orchestrates multiple steps of preinitiation complex assembly in vivo. Nucleic Acids Res. 2015;43: 9214–9231. doi: 10.1093/nar/gkv782 26240385 PMC4627066

[49] Tourigny JP, Schumacher K, Saleh MM, Devys D, Zentner GE. Architectural Mediator subunits are differentially essential for global transcription in Saccharomyces cerevisiae. Genetics. 2021;217. doi: 10.1093/genetics/iyaa042 33789343 PMC8045717

[50] Lynn Henry N, Campbell AM, Feaver WJ, Poon D, Anthony Weil P, Kornberg RD. TFIIF-TAF-RNA polymerase II connection. Genes Dev. 1994;8: 2868–2878. doi: 10.1101/gad.8.23.2868 7995524

[51] Rani PG, Ranish JA, Hahn S. RNA polymerase II (Pol II)-TFIIF and Pol II-mediator complexes: the major stable Pol II complexes and their activity in transcription initiation and reinitiation. Mol Cell Biol. 2004;24: 1709–20. doi: 10.1128/MCB.24.4.1709-1720.2004 14749386 PMC344180

[52] Tempe D, Vives E, Brockly F, Brooks H, De Rossi S, Piechaczyk M, et al. SUMOylation of the inducible (c-Fos:c-Jun)/AP-1 transcription complex occurs on target promoters to limit transcriptional activation. Oncogene. 2014;33: 921–927. doi: 10.1038/onc.2013.4 23396363

[53] Ryu H, Su D, Wilson-Eisele NR, Zhao D, López-Giráldez F, Hochstrasser M. The Ulp2 SUMO protease promotes transcription elongation through regulation of histone sumoylation. EMBO J. 2019 [cited 25 Jul 2019]. doi: 10.15252/embj.2019102003 31313851 PMC6694223

[54] Baptista T, Grünberg S, Minoungou N, Koster MJE, Timmers HTM, Hahn S, et al. SAGA Is a General Cofactor for RNA Polymerase II Transcription. Mol Cell. 2017;68: 130–143.e5. doi: 10.1016/j.molcel.2017.08.016 28918903 PMC5632562

[55] Psakhye I, Jentsch S. Protein group modification and synergy in the SUMO pathway as exemplified in DNA repair. Cell. 2012;151: 807–820. doi: 10.1016/j.cell.2012.10.021 23122649

[56] Downey M, Johnson JR, Davey NE, Newton BW, Johnson TL, Galaang S, et al. Acetylome profiling reveals overlap in the regulation of diverse processes by sirtuins, Gcn5, and esa1. Mol Cell Proteomics. 2015;14: 162–176. doi: 10.1074/mcp.M114.043141 25381059 PMC4288252

[57] Fang NN, Chan GT, Zhu M, Comyn SA, Persaud A, Deshaies RJ, et al. Rsp5/Nedd4 is the main ubiquitin ligase that targets cytosolic misfolded proteins following heat stress. Nat Cell Biol. 2014;16: 1227–1237. doi: 10.1038/ncb3054 25344756 PMC5224936

[58] Cramer P, Bushnell DA, Kornberg RD. Structural basis of transcription: RNA polymerase II at 2.8 ångstrom resolution. Science (80-). 2001;292: 1863–1876. doi: 10.1126/science.1059493 11313498

[59] Yudkovsky N, Ranish JA, Hahn S. A transcription reinitiation intermediate that is stabilized by activator. Nature. 2000;408: 225–229. doi: 10.1038/35041603 11089979

[60] Knop M, Siegers K, Pereira G, Zachariae W, Winsor B, Nasmyth K, et al. Epitope tagging of yeast genes using a PCR-based strategy: more tags and improved practical routines. Yeast. 1999;15: 963–972. doi: 10.1002/(SICI)1097-0061(199907)15:10B&lt;963::AID-YEA399&gt;3.0.CO;2-W [pii] 10407276

[61] Seufert W, Futcher B, Jentsch S. Role of a ubiquitin-conjugating enzyme in degradation of S- and M-phase cyclins. Nature. 1995;373: 78–81. doi: 10.1038/373078a0 7800043

[62] Svejstrup JQ, Petrakis TG, Fellows J. Purification of elongating RNA polymerase II and other factors from yeast chromatin. Methods Enzym. 2003;371: 491–498. doi: 10.1016/S0076-6879(03)71036-2 S0076687903710362 [pii] 14712723

[63] Ogami K, Richard P, Chen Y, Hoque M, Li W, Moresco JJ, et al. An Mtr4/ZFC3H1 complex facilitates turnover of unstable nuclear RNAs to prevent their cytoplasmic transport and global translational repression. Genes Dev. 2017;31: 1257–1271. doi: 10.1101/gad.302604.117 28733371 PMC5558927

[64] Chen ZA, Jawhari A, Fischer L, Buchen C, Tahir S, Kamenski T, et al. Architecture of the RNA polymerase II–TFIIF complex revealed by cross-linking and mass spectrometry. EMBO J. 2010;29: 717–726. doi: 10.1038/emboj.2009.401 20094031 PMC2810376

