# Supplementary figures and images for: Dynamic sumoylation of promoter-bound general transcription factors facilitates transcription by RNA polymerase II
Source: PLoS Genet. 2021 Sep 29;17(9):e1009828. doi: 10.1371/journal.pgen.1009828 (PMC8505008; doi:10.1371/journal.pgen.1009828)

Figure S1

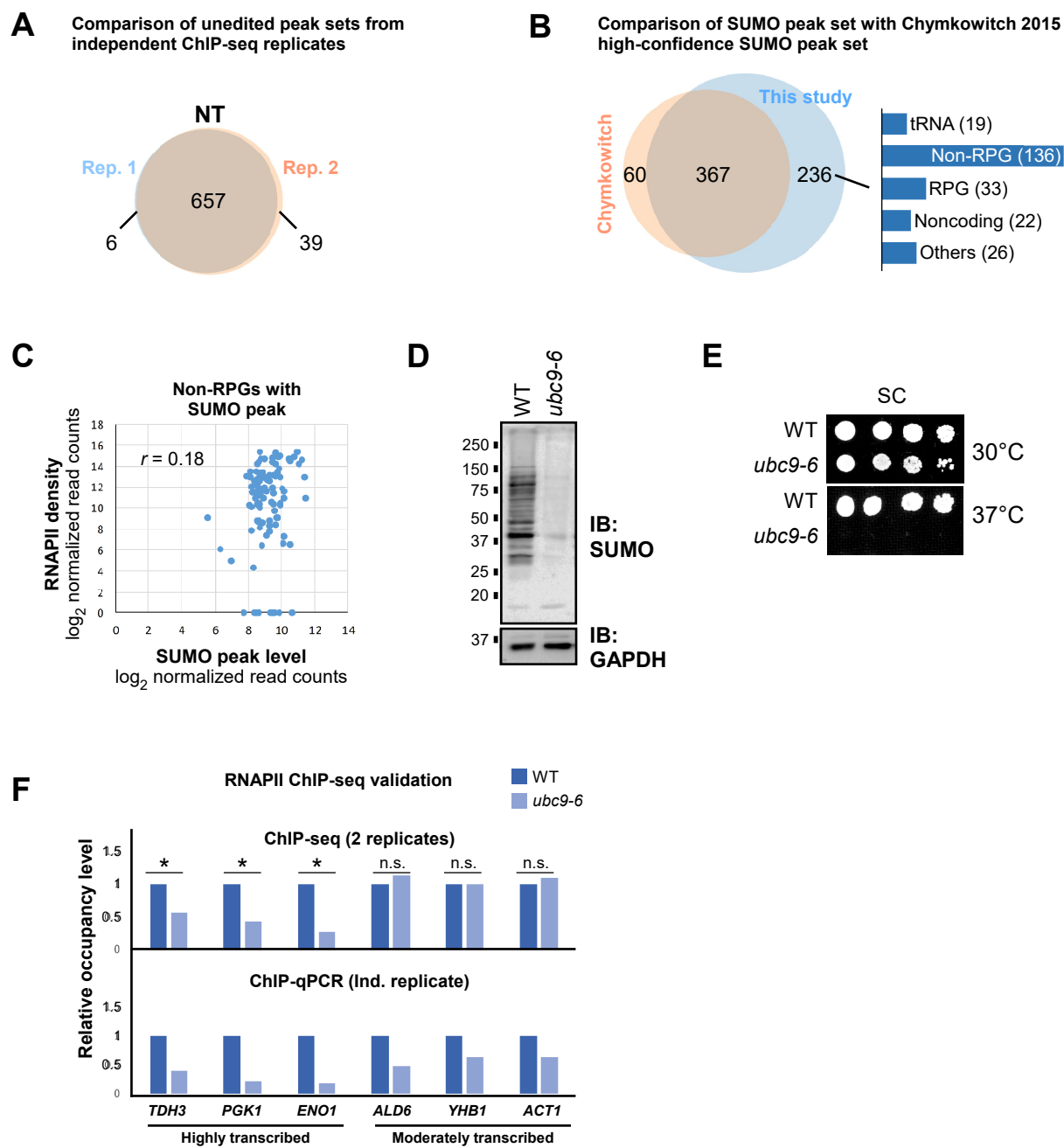

Supplement: S1 Fig — (A) Venn diagram showing comparison of unedited peak sets from the two independent SUMO ChIP-seq replicates. (B) Venn diagram showing comparison of the high stringency SUMO ChIP-seq peak set from this study and the high stringency Flag-SUMO ChIP-seq peak set from a previous study [40]. The current study identified 236 high stringency SUMO peaks that were not identified in the previous study, 136 of which are associated with non-RPGs. (C) Levels of SUMO peaks do not correlate with transcription level, as approximated by RNAPII densities. Scatter plot of SUMO peak level versus RNAPII density for 111 non-RPGs with a unique SUMO peak. Pearson coefficient (r) implies no significant correlation. (D) The ubc9-6 strain has dramatically reduced SUMO conjugation levels. SUMO and GAPDH immunoblots of lysates prepared from ubc9-6 and its parental strain (WT; W303a). All SUMO-peaks at non-RPGs were dramatically reduced in ubc9-6 (see Fig 2C and 2D). (E) Spot assays examining growth of the ubc9-6 strain on SC medium at the permissive temperature (30°), in which experiments in this study were performed, or at the non-permissive temperature of 37°C. (F) Validation of RNAPII ChIP-seq. Occupancy values obtained from DiffBind analysis of the two ChIP-seq replicates for WT and ubc9-6 strains across ORFs of selected genes is shown at top. An independent RNAPII ChIP was performed in the same strains and qPCR analysis of the promoter region of the same genes is shown at bottom. Genes analyzed are a selection of SUMO peak-containing genes that include those with high RNAPII density (TDH3, PGK1, ENO1) and modest RNAPII density (ALD6, YHB1, ACT1), as determined by our ChIP-seq analyses. Asterisks (*) indicate genes with significantly different RNAPII occupancy levels in the two strains (FDR < 0.05) according to the DiffBind analysis (see S5 Table for details). (PDF) [file pgen.1009828.s001.pdf]

Figure S2

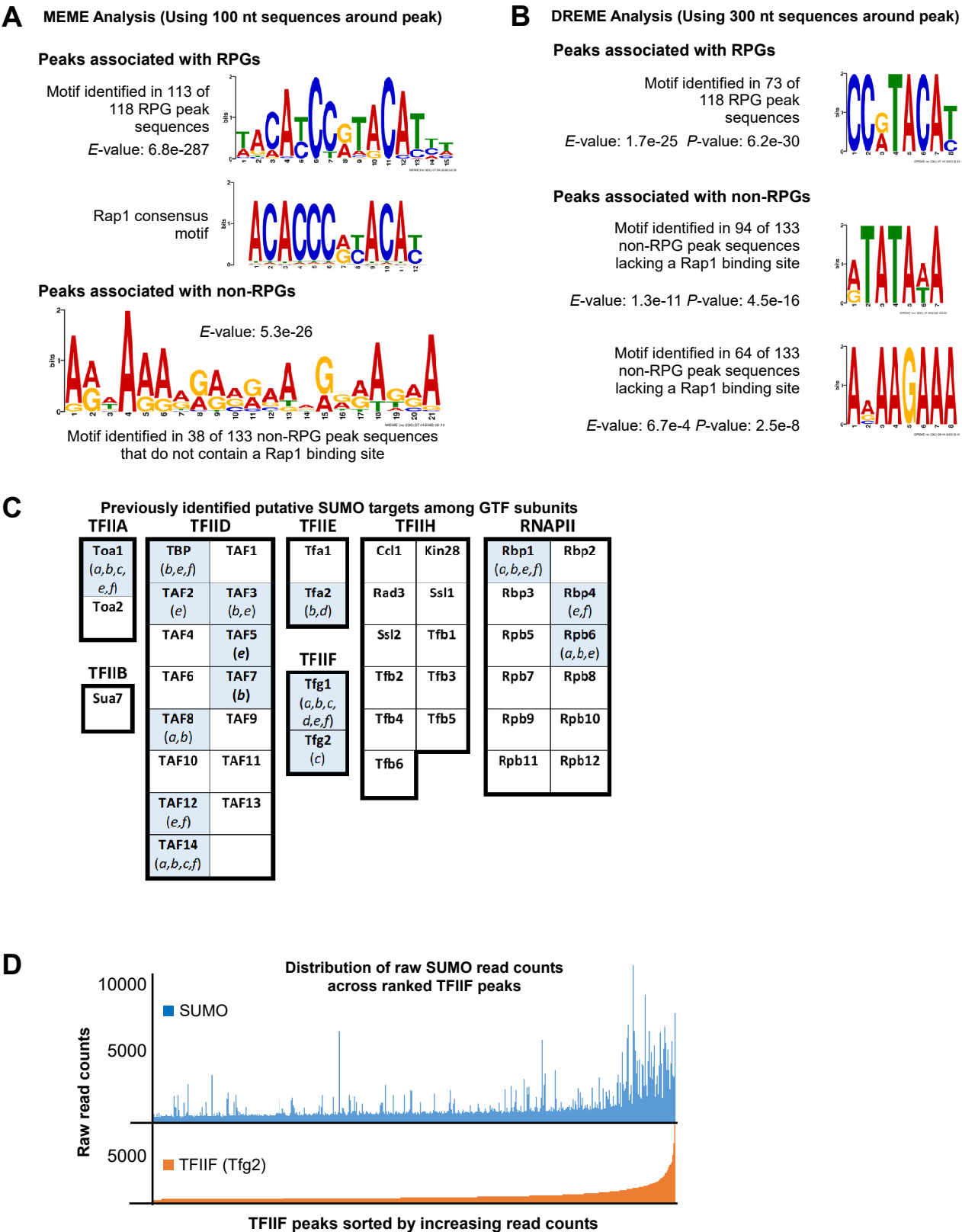

Supplement: S2 Fig — (A) Motif analysis of SUMO peak-containing sequences. MEME analysis, for identifying novel, ungapped, recurring patterns [47], was used with 100-nt sequences encompassing 118 RPG-associated SUMO peaks or 133 SUMO peaks associated with non-RPGs that do not contain a known Rap1 binding site. The top motif identified for the RPG set is shown, which matches the known Rap1 binding site consensus (also shown). Only one motif was identified for the non-RPG set, as shown. (B) Motif analysis using DREME, from the MEME suite of tools, was applied to identify short (8-nt or less) motifs that are relatively enriched, using 300-nt sequences encompassing the RPG or non-RPG SUMO peak sets described in A. The top result is shown for the RPG set, which matches the Rap1 binding motif (see A). Both significant results produced for the non-RPG set are shown, with the top result indicating that 94 of the 133 sequences contain a TATA box element (consensus TATAWAWR). (C) Multiple subunits of GTFs and RNAPII are putative SUMO targets (shaded in blue), based on published proteomics analyses. Note that TAF14, also known as Tfg3, is also considered a subunit of yeast TFIIF. References are as follows: a, [30]; b, [31]; c, [32]; d, [33]; e, [8]; f, [10]. Note that only Tfg1 has been identified all six studies. (D) Genomic sites with high levels of TFIIF generally show high levels of SUMO. TFIIF ChIP-seq peaks across the genome were identified (for its Tfg2 subunit) and plotted by increasing raw read count values (based on data obtained from GEO database accession number GSM4319120). Raw read counts from our SUMO ChIP-seq analysis at each of the corresponding TFIIF peaks were determined and plotted above. (PDF) [file pgen.1009828.s002.pdf]

Figure S3

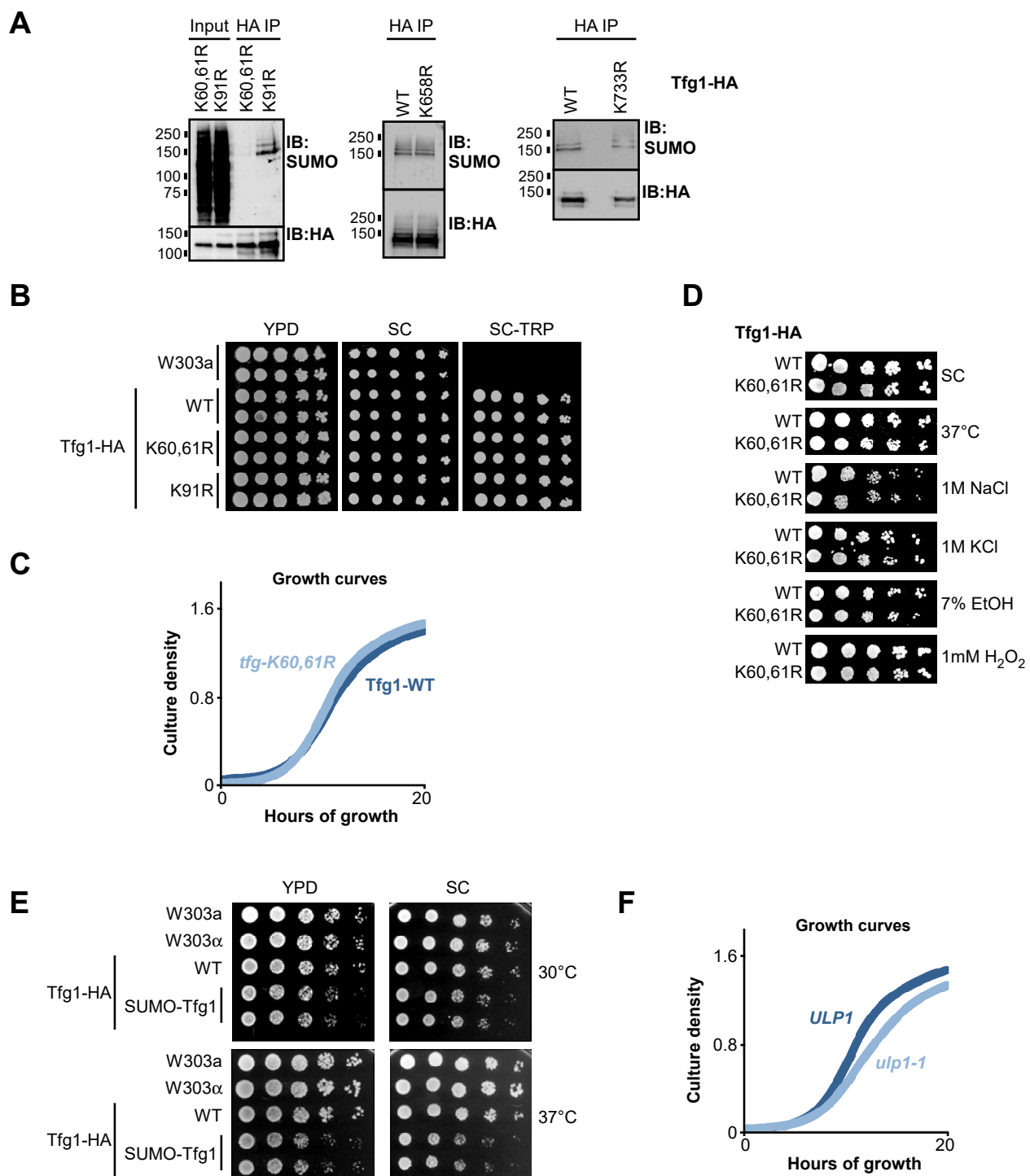

Supplement: S3 Fig — (A) Arg substitution of Lys 60,61, but not Lys 91, 658, or 733, significantly impairs sumoylation of Tfg1. Tfg1-HA-expressing strains with the indicated Lys-to-Arg substitutions, or unmodified (WT), were used in HA IP experiments followed by SUMO and HA immunoblots to examine the effects of the mutations on Tfg1 sumoylation levels. (B) Impairing Tfg1 sumoylation is not sufficient to affect growth. Strains expressing Tfg1-HA in its wild-type form (WT), or with K60,61R or K91R mutations, and the parental, unmodified lab strain W303a, were used in a spot assay, comparing growth on rich medium (YPD), synthetic medium (SC), or synthetic medium lacking tryptophan (SC-TRP). Strains expressing HA-tagged Tfg1 are marked with the Kluyveromyces lactis TRP1 gene. (C) Liquid cultures of WT or K60,61R version of Tfg1-HA strains were prepared at an absorbance (595 nm) of ~0.2, then culture absorbance measurements were taken over a 20 h period using an accuSkan absorbance microplate reader (Fisherbrand). Culture density values are arbitrary. Average of three experiments is shown; error bars representing standard deviation are small and obscured by the thickness of the curves. (D) Spot assays were performed to compare growth of strains expressing WT or K60,61R forms of Tfg1-HA on SC medium in normal conditions or in the presence of the indicated stressors. Normal yeast growth temperature is 30°C. (E) Spot assays were performed on YPD or SC medium at 30°C or 37°C using unmodified lab strains W303a and W303α, a strain expressing WT Tfg1-HA, and a strain expressing Tfg1-HA with an N-terminal fusion to the yeast SUMO peptide (Smt3 residues 1–96, lacking the protease-targeting C-terminal GG motif). (F) Liquid growth curves were generated for ULP1 and ulp1-mt strains, as in S3C Fig. (PDF) [file pgen.1009828.s003.pdf]

Figure S4

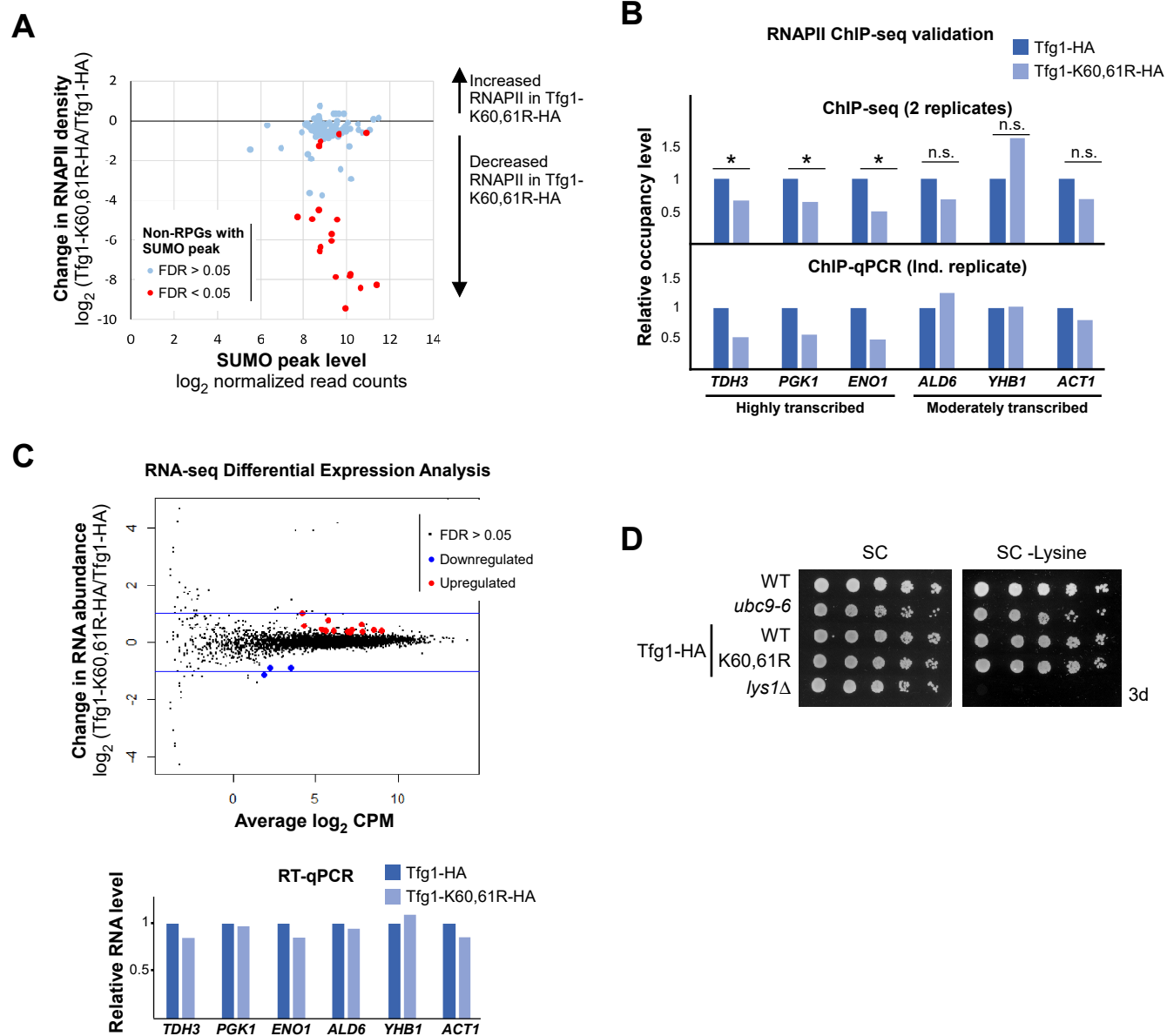

Supplement: S4 Fig — (A) Scatterplot showing changes in RNAPII densities for non-RPGs associated with unique SUMO peaks in Tfg1-HA versus Tfg1-K60,61R-HA strains, sorted horizontally by SUMO peak levels. Genes showing a significant difference, as determined by differential binding analysis with DiffBind, are represented by red dots. (B) Validation of RNAPII ChIP-seq. Occupancy values obtained from DiffBind analysis of the two ChIP-seq replicates for Tfg1-HA and Tfg1-K60,61R-HA strains across ORFs of selected genes is shown at top. An independent RNAPII ChIP was performed in the same strains and qPCR analysis of the promoter region of the same genes is shown at bottom. Genes analyzed are a selection of SUMO peak-containing genes that include those with high RNAPII density (TDH3, PGK1, ENO1) and modest RNAPII density (ALD6, YHB1, ACT1), as determined by our ChIP-seq analyses. Asterisks (*) indicate genes with significantly different RNAPII occupancy levels in the two strains (FDR < 0.05) according to the DiffBind analysis (see S6 Table for details). (C) RNA-seq analysis was performed in Tfg1-HA and Tfg1-K60,6R-HA strains, and differential expression analysis (using edgeR) is plotted, as log2 of the ratio of expression in the two strains, sorted horizontally by average log2 expression (in counts per million, CPM). Genes showing significantly higher RNA abundance in Tfg1-K60,61R-HA are represented with red dots, and those showing significantly lower RNA abundance in that strain are shown in blue. See S13 Table for results of RNA-seq analysis. At bottom, an independent set of RNAs was obtained from the same strains and RT-qPCR was performed on a selection of genes, which also shows no significant difference in steady-state RNA levels. (D) Reduced global sumoylation or impaired Tfg1 sumoylation does not result in Lys auxotrophy. Spot assay comparing growth of indicated strains on SC medium and SC medium lacking lysine. (PDF) [file pgen.1009828.s004.pdf]
