# Supplementary material for: Dynamic sumoylation of promoter-bound general transcription factors facilitates transcription by RNA polymerase II
Source: PLoS Genet. 2021 Sep 29;17(9):e1009828. doi: 10.1371/journal.pgen.1009828 (PMC8505008; doi:10.1371/journal.pgen.1009828)
Supplement: S1 Table — (PDF) [file pgen.1009828.s005.pdf]

S1 Table. Yeast strains used in this study

Name	Code	Background/ Parent strain	Genotype	Source
W303a			*MAT a ura3-52 trp1Δ2 leu2-3_112 his3-11 ade2-1 can1-100*	Dharmacon
W303α			*MAT α ura3-52 trp1Δ2 leu2-3_112 his3-11 ade2-1 can1-100*	Dharmacon
ubc9-6	D370	W303a	*ubc9-1:Tadh1:TRP1:ubc9-1*	This study
Toa1-HA	YJB001A	W303a	*TOA1-6HA::K. lactis TRP1*	This study
Taf7-HA	YVS001A	W303a	*TAF7-6XHA::K. lactis TRP1*	This study
Taf8-HA	YVS002A	W303a	*TAF8-6XHA::K. lactis TRP1*	This study
Tfg1-HA	YJB002A	W303a	*TFG1-6XHA::K. lactis TRP1*	This study
Tfg2-HA	YJB004A	W303a	*TFG2-6XHA::K. lactis TRP1*	This study
Tfg2-9HA	YJB004C	W303a	*TFG2-9XHA::K. lactis TRP1*	This study
Tfg3-HA	YRB005C	W303a	*TFG3-6XHA::K. lactis TRP1*	This study
Tfg1-K60,61R	YRB013A	W303a	*tfg1-K60,61R-6XHA::K. lactis TRP1* with inadvertent N462Q substitution	This study
ULP1	MH1006		*leu2-3,112 ura3-52 trp1-289 ade2Δ ade3Δ lys1::kanMX4*	Martine Heude [1]
ulp1-mt	MH1018	MH1006	*ulp1-I615N*	Martine Heude [1]
ULP1 / Tfg1-HA	YYD002D	MH1006	*ULP1 TFG1-6XHA::K. lactis TRP1*	This study
ulp1-mt / Tfg1-HA	YYD003A	MH1018	*ulp1-I615N TFG1-6XHA::K. lactis TRP1*	This study
SUMO-Tfg1-HA	YJBM040	W303	*SMT3(1-96ΔGG)-TFG1-6HA::K. lactis TRP1*	This study
mSUMO-Tfg1-HA	YJBM043	W303	*smt3-KallR-(1-96ΔGG)-TFG1-6HA::K. lactis TRP1*	This study
Tfg1-K91R	YRB015C	W303a	*tfg1-K91R-6XHA::K. lactis TRP1*	This study
Tfg1-K658R	YJB003C	W303a	*tfg1-K658R-6XHA::K. lactis TRP1*	This study
Tfg1-K733R		W303a	*tfg1-K733R-6XHA::K. lactis TRP1*	This study

REFERENCE

1. Soustelle C, Vernis L, Freon K, Reynaud-Angelin A, Chanet R, Fabre F, et al. A new Saccharomyces cerevisiae strain with a mutant Smt3-deconjugating Ulp1 protein is affected in DNA replication and requires Srs2 and homologous recombination for its viability. Mol Cell Biol. 2004;24: 5130–5143. doi:10.1128/MCB.24.12.5130-5143.2004
