# Supplementary material for: Dynamic sumoylation of promoter-bound general transcription factors facilitates transcription by RNA polymerase II
Source: PLoS Genet. 2021 Sep 29;17(9):e1009828. doi: 10.1371/journal.pgen.1009828 (PMC8505008; doi:10.1371/journal.pgen.1009828)
Supplement: S2 Table — (PDF) [file pgen.1009828.s006.pdf]

S2 Table. Peak set statistics for SUMO ChIP-seq

Initial peak set	702 peaks	Used DiffBind to generate a consensus peak set from two independent replicates of SUMO ChIP.
High-stringency peak set	603	Eliminated peaks that are not discernable in both WT replicates (relative to adjacent areas) through qualitative visual inspection of each peak.
**Of the 603 high-stringency peaks:**		
tRNA	257	Peaks associated with one or two tRNA-encoding genes
Non-RPG	147	Peaks associated with promoter of one or two protein-coding genes that are not ribosomal protein genes (RPGs)<br>• 171 genes are associated with these peaks<br>• 123 of these peaks are associated with a single gene<br>• 24 of these peaks are associated with two genes
	133	Non-RPG peaks that do not overlap with a Rap1 peak (from Rhee and Pugh, 2011)<br>• 155 genes are associated with these peaks<br>• 111 of these peaks are associated with a single gene<br>• 22 of these peaks are associated with two genes
RPG	118	Peaks associated with promoter of one or two ribosomal protein genes<br>• 123 RPGs are associated these peaks<br>• 113 of these peaks are associated with a single RPG<br>• 5 of these peaks are associated with two RPGs
Noncoding	43	Peaks associated with one or more noncoding RNA genes
Others	38	Peaks not associated with known genes, or associated with two different types of genes
