# Supplementary material for: Dynamic sumoylation of promoter-bound general transcription factors facilitates transcription by RNA polymerase II
Source: PLoS Genet. 2021 Sep 29;17(9):e1009828. doi: 10.1371/journal.pgen.1009828 (PMC8505008; doi:10.1371/journal.pgen.1009828)
Supplement: S3 Table — (PDF) [file pgen.1009828.s007.pdf]

S3 Table. Statistics for RNAPII ChIP-seq and RNA-seq in Tfg1-HA vs. Tfg1-K60,61R strains

**RNAPII ChIP-seq (See Table S12)**		
ORFs examined	5990	RNAPII density was determined at 5990 ORFs.
	2838	ORFs showed significantly **less** RNAPII density in the Tfg1-K60,61R-HA strain than in the Tfg1-HA strain<br>• 36 of these are non-RPGs that contain a promoter-associated SUMO peak.
	8	ORFs showed significantly **higher** RNAPII density in the Tfg1-K60,61R-HA strain than in the Tfg1-HA strain<br>• None of these are non-RPGs that contain a SUMO peak.
**RNA-seq (See Table S13)**		
Genes examined	6020	RNA levels were determined at 6020 genes after polyA-enrichment of total RNA.
	2	Genes showed significantly **lower** levels of RNA in the Tfg1-K60,61R-HA strain than in the Tfg1-HA strain
	14	Genes showed significantly **higher** levels of RNA in the Tfg1-K60.61R-HA strain than in the Tfg1-HA strain
