# Supplementary material for: Dynamic sumoylation of promoter-bound general transcription factors facilitates transcription by RNA polymerase II
Source: PLoS Genet. 2021 Sep 29;17(9):e1009828. doi: 10.1371/journal.pgen.1009828 (PMC8505008; doi:10.1371/journal.pgen.1009828)
Supplement: S4 Table — (PDF) [file pgen.1009828.s008.pdf]

S4 Table. Analysis details for SUMO ChIP-seq

**SUMO ChIP-seq**	
Samples and conditions	Two independent replicates were prepared from cultures grown in SC medium at 30°C. Inputs and SUMO IPs were sequenced.<br>1 – WT (W303a): untreated<br>2 – *ubc9-6*: untreated
Library synthesis	NEBNext Ultra II DNA library prep kit (New England Biolabs)
Sequencing	Illumina HiSeq 2500; Paired-end reads; 2x 126 nt; 10 million reads/sample
Quality control	FastQC (0.11.5)
Trimming	Trim Galore (0.4.4_dev)<br>Cutadapt (2.3)<br>Quality Phred score cutoff: 25, Adapter sequence: 'AGATCGGAAGAGC', Minimum required adapter overlap (stringency): 5 bp, Minimum required sequence length for both reads before a sequence pair gets removed: 40 bp, All sequences trimmed by 6 bp from their 5' end.
Genome alignment	Bowtie2 (2.3.5) with sacCer3 reference genome
Peak calling	MACS (2.1.1.20160309)<br>***Parameters:*** paired-end; input as control; narrow peaks; effective genome size: 1.2e7; *q*-value cut-off: 0.05
Differential binding analysis	DiffBind (2.10.0)<br>***Parameters:*** Defaults with minMembers=2 for dba.contrast; see notes below
Peak analysis and annotation	ChIPpeakAnno (3.2.0) from Bioconductor<br>***Parameters:*** TxDb.Scerevisiae.UCSC.sacCer3.sgdGene genome annotation package was used, but each annotation was confirmed visually using IGV.
Composite (“meta-gene”) plot	computeMatrix tool from deepTools (3.5.0)<br>***Parameters:*** Bowtie2-produced BAM files for Replicate 1 (WT and *ubc9-6*) were converted to BED format and analyzed with a custom-made BED regions file containing only SUMO peak-containing non-RPGs .
Motif analysis	MEME suite (5.1.0) tools MEME and DREME were used with sequences surrounding peaks obtained with ChIPpeakAnno tool peakWithSequences.
GO analysis	Gene names for the SUMO peak list of non-RPGs (Table S9) were analyzed by the GO Term Finder (0.86) on the Saccharomyces Genome Database (yeastgenome.org) with default parameters.
Notes	To establish SUMO peak list (Table S9):<br>• WT and *ubc9-6* sets were compared to identify “differentially bound” sites with DiffBind, but all peaks identified in the WT set were considered. Some SUMO peaks, particularly those associated with tRNAs, are stable, even with low sumoylation levels in *ubc9-6*.

S4 Table. Analysis details for SUMO ChIP-seq

	• Each peak was visually inspected on IGV to validate and classify by associated gene type and confirm annotations attributed by ChIPpeakAnno.<br>• DiffBind-generated “concentration” values from the WT set was taken as log_2 normalized ChIP read counts with control read counts subtracted.
