# Supplementary material for: Dynamic sumoylation of promoter-bound general transcription factors facilitates transcription by RNA polymerase II
Source: PLoS Genet. 2021 Sep 29;17(9):e1009828. doi: 10.1371/journal.pgen.1009828 (PMC8505008; doi:10.1371/journal.pgen.1009828)
Supplement: S5 Table — (PDF) [file pgen.1009828.s009.pdf]

S5 Table. Analysis details for RNAPII ChIP-seq in WT vs. *ubc9-6* strains

**RNAPII ChIP-seq in WT and *ubc9-6* strains**	
Samples and conditions	Two independent replicates were prepared from cultures grown in SC medium at 30°C. Inputs and 8WG16 (Rpb1 antibody) IPs were sequenced.<br>1 – WT (W303a)<br>2 – *ubc9-6*
Library synthesis	NEBNext Ultra II DNA library prep kit (New England Biolabs)
Sequencing	Illumina HiSeq 2500; Paired-end reads; 2x 126 nt; 10 million reads/sample
Quality control	FastQC (0.11.8)
Trimming	Trim Galore (0.4.4_dev)<br>Cutadapt (1.18)<br>Quality Phred score cutoff: 25, Adapter sequence: 'AGATCGGAAGAGC', Minimum required adapter overlap (stringency): 5 bp, Minimum required sequence length for both reads before a sequence pair gets removed: 40 bp, All sequences trimmed by 6 bp from their 5' end.
Genome alignment	Bowtie2 (2.3.5) with sacCer3 reference genome
Peak calling	MACS (2.1.1.20160309)<br>***Parameters:*** paired-end; input as control; broad peaks; effective genome size: 1.2e7; *q*-value cut-off: 0.1
Differential binding analysis	DiffBind (2.8.0)<br>***Parameters:*** minMembers=2 for dba.contrast; th=1 for dba.report; see notes below
Peak analysis and annotation	ChIPpeakAnno (3.2.0) from Bioconductor<br>***Parameters:*** TxDb.Scerevisiae.UCSC.sacCer3.sgdGene genome annotation package was used and the closest feature to the middle of each peak was used for annotation.
Notes	To determine RNAPII density at each ORF:<br>• DiffBind was applied to the four samples using a pre-defined peak-set that corresponds to ORF regions of all protein-coding genes. The “th=1” parameter was applied to determine the “concentration” (log_2 normalized ChIP read counts with control read counts subtracted) of RNAPII at all ORFs in all four conditions. Here, these are referred to as RNAPII densities.<br>• Table S11 shows RNAPII densities for all genes in the four samples, with pairwise comparisons for WT vs. *ubc9-6* samples.<br>• For this study, only data relevant to the analysis of SUMO-containing non-RPGs are shown. Full analysis of this experiment will be presented elsewhere.
