# Supplementary material for: Dynamic sumoylation of promoter-bound general transcription factors facilitates transcription by RNA polymerase II
Source: PLoS Genet. 2021 Sep 29;17(9):e1009828. doi: 10.1371/journal.pgen.1009828 (PMC8505008; doi:10.1371/journal.pgen.1009828)
Supplement: S6 Table — (PDF) [file pgen.1009828.s010.pdf]

S6 Table. Analysis details for RNAPII ChIP-seq in Tfg1-HA vs. Tfg1-K60,61R strains

**RNAPII ChIP-seq in Tfg1-HA and Tfg1-K60,61R strains**	
Samples and conditions	Two independent replicates were prepared from cultures grown in SC medium at 30°C. Inputs and 8WG16 (Rpb1 antibody) IPs were sequenced.<br>1 – Tfg1-HA<br>2 – Tfg1-K60,61R
Library synthesis	NEBNext Ultra II DNA library prep kit (New England Biolabs)
Sequencing	Illumina HiSeq 2500; Paired-end reads; 2x 126 nt; 10 million reads/sample
Quality control	FastQC (0.11.9)
Trimming	TrimGalore (0.6.6)<br>***Parameters:*** paired, length 40, stringency 5, illumina –q 25, clipped 6 bp from 5' end
Genome alignment	Bowtie2 (2.3.5.1) with sacCer3 reference genome
Peak calling	MACS (2.2.7.1)<br>***Parameters:*** paired-end; input as control; broad region calling; effective genome size 1.2e7; *q*-value cut-off: 0.1
Differential binding analysis	DiffBind (2.16.0)<br>***Parameters:*** minMembers=2 for dba.contrast; th=1 for dba.report; see notes below
Peak analysis and annotation	ChIPpeakAnno (3.22.0) from Bioconductor<br>***Parameters:*** TxDb.Scerevisiae.UCSC.sacCer3.sgdGene genome annotation package was used and the closest feature to the middle of each peak was used for annotation.
Notes	To determine RNAPII density at each ORF:<br>• DiffBind was applied to the four samples using a pre-defined peak-set that corresponds to ORF regions of all protein-coding genes. The "th=1" parameter was applied to determine the "concentration" (log_2 normalized ChIP read counts with control read counts subtracted) of RNAPII at all ORFs in both conditions. Here, these are referred to as RNAPII densities.<br>• Table S12 shows RNAPII densities at all genes in the two samples.
