# Supplementary material for: Dynamic sumoylation of promoter-bound general transcription factors facilitates transcription by RNA polymerase II
Source: PLoS Genet. 2021 Sep 29;17(9):e1009828. doi: 10.1371/journal.pgen.1009828 (PMC8505008; doi:10.1371/journal.pgen.1009828)
Supplement: S7 Table — (PDF) [file pgen.1009828.s011.pdf]

S7 Table. Analysis details for RNA-seq in Tfg1-HA vs. Tfg1-K60,61R-HA strains

**RNA-seq in Tfg1-HA and Tfg1-K60,61R-HA strains**	
Samples and conditions	Two independent replicates were prepared from cultures grown in SC medium at 30°C.<br>1 – Tfg1-HA<br>2 – Tfg1-K60,61R
Library synthesis	NEB Ultra II directional mRNA library prep (PolyA enrichment)
Sequencing	Illumina HiSeq 2500; Paired-end reads; 2x 126 nt; 30 million reads/sample
Quality control	FastQC (0.11.9)
Genome alignment	HISAT2 (2.1.0)<br>***Parameters:*** Default options; UCSC annotaion file: sacCer3.ncbiRefSeq.gtf
Transcript counting	Transcripts counted using featureCounts from Subread (2.0.0)<br>***Parameters:*** Default options; -p (paired)
Differential expression analysis	edgeR (3.32.1)<br>***Parameters:*** Library sizes were normalized using calcNormFactors; dispersions were estimated with estimateDisp with robust argument; likelilhood ratio tests for differential expression were performed with glmFit and glmLRT.
