# Supplementary material for: Dynamic sumoylation of promoter-bound general transcription factors facilitates transcription by RNA polymerase II
Source: PLoS Genet. 2021 Sep 29;17(9):e1009828. doi: 10.1371/journal.pgen.1009828 (PMC8505008; doi:10.1371/journal.pgen.1009828)
Supplement: S8 Table — (PDF) [file pgen.1009828.s012.pdf]

S8 Table. Sequences of oligonucleotides used in this study

Gene	Oligonucleotide sequence(s)
Primers for qPCR analysis of ChIP samples	
TDH1 promoter	TGACCAAAACTGGAGTCTCG
	TGCAAGAGAGAGAATAGAACTG
PDC1 (CDC19) promoter	CTCCTTGCAATCAGATTTGG
	TTGCGTGAGGTTATGAGTAG
PYK1 promoter	CTCCTTGCAATCAGATTTGG
	TTGCGTGAGGTTATGAGTAG
RPS20 promoter	CGCGACTAGCCTCAGAGATT
	GCTGAGCTTGAATGAAATAACCC
TDH3 promoter	TTAACGGTTTCGGTAGAATCGG
	AACAACTTCGACGTTTGGTCTA
PGK1 promoter	TCCAGAGCAAAGTTCGTTCG
	TGTTGTTGTCACACGATTCGG
ENO1 promoter	TGGAAACCTTGTCACCTCAC
	ATCGTGGAGATCATGTGTGC
ALD6 promoter	AGCTCAAACAGCGATTTAACGG
	GAACTTGTTAAACACGCCAGG
YHB1 promoter	CAAGTACAAGACTACTGATG
	CGGCTAGCATAATGAATAAAG
ACT1 promoter	AGTCTCATGTACTAACATCGATTGC
	CCAAAGCAGCAACCTCTAAAC
Untranscribed region of Chr. V	CATTATCCGTAACGCCACTTT
	CGATCTTAGTTCCAATGGTGAAA
Primers for qPCR analysis of RT samples	
TDH3	CTGTCCACTCTTTGACTGCTA
	CTCTCCAGTCCTTGTGGGAT
PGK1	CGGTTTCGACTTGCCACAAC
	TGGTTGGGTTCTCCAAAGCC
ENO1	CGTCTGCATCCTCCATTGGG
	CATTGGGCAGCTTGATAGGC
ALD6	ATGTTGGCTTGGAAGATCGC
	TTGGTCAAAGCAGCACCAAC
YHB1	CAAGCAAATTGGCCACAAGC
	AATTGCTTGGTAAGCCTCGC
ACT1	GAAATCACCGCTTTGGCTCC
	GAACCACCAATCCAGACGGA
25S rRNA	TCTAGCATTCAAGGTCCCATTC
	CCCTTAGGACATCTGCGTTATC
